# Blocking glutamate mGlu_5_ receptors with the negative allosteric modulator CTEP improves disease course in SOD1^G93A^ mouse model of amyotrophic lateral sclerosis

**DOI:** 10.1111/bph.15515

**Published:** 2021-06-29

**Authors:** Marco Milanese, Tiziana Bonifacino, Carola Torazza, Francesca Provenzano, Mandeep Kumar, Silvia Ravera, Arianna Roberta Zerbo, Giulia Frumento, Matilde Balbi, T. P. Nhung Nguyen, Nadia Bertola, Sara Ferrando, Maurizio Viale, Aldo Profumo, Giambattista Bonanno

**Affiliations:** ^1^ Department of Pharmacy, Pharmacology and Toxicology Unit University of Genoa Genoa Italy; ^2^ Inter‐University Center for the Promotion of the 3Rs Principles in Teaching & Research (Centro 3R) Genoa Italy; ^3^ Present address: Department of Neurodegenerative Diseases Hertie Institute for Clinical Brain Research, University of Tübingen and German Center for Neurodegenerative Diseases (DZNE) Tübingen Germany; ^4^ Department of Experimental Medicine University of Genoa Genoa Italy; ^5^ Department of Earth, Environmental and Life Science University of Genoa Genoa Italy; ^6^ IRCCS Ospedale policlinico San Martino Genoa Italy

**Keywords:** 2‐chloro‐4‐((2,5‐dimethyl‐1‐(4‐(trifluoromethoxy)phenyl)‐1*H*‐imidazol‐4‐yl)ethynyl)pyridine (CTEP), amyotrophic lateral sclerosis (ALS), in vivo pharmacological treatment, metabotropic glutamate receptor 5 (mGlu5 receptor), SOD1^G93A^ mice

## Abstract

**Background and Purpose:**

The pathogenesis of amyotrophic lateral sclerosis (ALS) is not fully clarified, although excessive glutamate (Glu) transmission and the downstream cytotoxic cascades are major mechanisms for motor neuron death. Two metabotropic glutamate receptors (mGlu_1_ and mGlu_5_) are overexpressed in ALS and regulate cellular disease processes. Expression and function of mGlu_5_ receptors are altered at early symptomatic stages in the SOD1^G93A^ mouse model of ALS and knockdown of mGlu5 receptors in SOD1^G93A^ mice improved disease progression.

**Experimental Approach:**

We treated male and female SOD1^G93A^ mice with 2‐chloro‐4‐((2,5‐dimethyl‐1‐(4‐(trifluoromethoxy)phenyl)‐1*H*‐imidazol‐4‐yl)ethynyl)pyridine (CTEP), an orally available mGlu_5_ receptor negative allosteric modulator (NAM), using doses of 2 mg·kg^−1^ per 48 h or 4 mg·kg^−1^ per 24 h from Day 90, an early symptomatic disease stage. Disease progression was studied by behavioural and histological approaches.

**Key Results:**

CTEP dose‐dependently ameliorated clinical features in SOD1^G93A^ mice. The lower dose increased survival and improved motor skills in female mice, with barely positive effects in male mice. Higher doses significantly ameliorated disease symptoms and survival in both males and females, females being more responsive. CTEP also reduced motor neuron death, astrocyte and microglia activation, and abnormal glutamate release in the spinal cord, with equal effects in male and female mice. No differences were also observed in CTEP access to the brain.

**Conclusion and Implications:**

Our results suggest that mGlu_5_ receptors are promising targets for the treatment of ALS and highlight mGlu5 receptor NAMs as effective pharmacological tools with translational potential.

**Abbreviations**
3,5‐DHPG: (*S*)‐3,5‐dihydroxyphenylglycine
CTEP: 2‐chloro‐4‐((2,5‐dimethyl‐1‐(4‐(trifluoromethoxy)phenyl)‐1*H*‐imidazol‐4‐yl)ethynyl)pyridine
EAAT2: excitatory amino acid transporter‐2
GFAP: glial fibrillary acidic protein
IBA‐1: ionized calcium‐binding adapter molecule
MN: motor neuron
NAM: negative allosteric modulator

**What is already known**

- Amyotrophic lateral sclerosis (ALS) is a multifactorial and multicellular fatal neurodegenerative disease lacking effective treatments.
- Excessive glutamate transmission, its downstream excitotoxic events and mGlu_5_ receptors are involved in ALS progression.

**What does this study add**

- Treatment with CTEP improves survival and the clinical course of disease, predominantly in SOD1^G93A^ females.
- CTEP administration decreased motor neuron death, astrogliosis, microgliosis and excessive glutamate transmission in SOD1^G93A^ mice.

**What is the clinical significance**

- Selective inhibition of mGlu_5_ receptor activity provides an effective approach to the treatment of ALS.
- This preclinical evidence supports mGlu5 receptor negative allosteric modulators as new tools for ALS cure.

## 1 INTRODUCTION

Amyotrophic lateral sclerosis (ALS) is the most common adult‐onset neuromuscular disease, with an unescapable fatal outcome. ALS causes selective death of motor neurons (MNs) in the motor cortex, brainstem and spinal cord (Eisen, 2009), leading to muscle wasting, weakness and spasticity. No effective cures are available for ALS, and the approved drugs show very limited benefit in patients (Abe et al., 2017; Lu et al., 2016). The development of effective therapies is thus urgently required, but it is hampered by the lack of the complete understanding of neuronal and non‐neuronal mechanisms underlying the MN damage and death (Verber et al., 2019).

Both sporadic (sALS) and familial (fALS) ALS are known and exhibit similar pathological traits. At least 15 genes have been associated with fALS (Chiò et al., 2014), implying that multiple causes may contribute to the disease (Ferraiuolo et al., 2011; Le Gall et al., 2020). Moreover, robust evidence of the involvement of astrocytes, microglia and oligodendrocytes in ALS, supports the view that this is not a cell‐autonomous disease (Filipi et al., 2020).

High levels of glutamate and hyperexcitability play a fundamental role in ALS (Shaw et al., 1995; Van Den Bosch et al., 2006). The elevated levels of glutamate are sustained by the reduced expression of the glutamate transporter, excitatory amino acid transporter‐2 (EAAT2) (Rothstein et al., 1992, 1995), and by abnormal release of the excitatory amino acid, due to plastic changes at glutamatergic nerve terminals and glial perisynaptic processes (Bonifacino et al., 2016; Milanese et al., 2010, 2011, 2015; Raiteri et al., 2004). Possible targets of excessive glutamate are presynaptic and postsynaptic ionotropic and metabotropic glutamate (mGlu) receptors. mGlu receptors comprise eight subtypes, divided into three groups. Group I includes mGlu_1_ and mGlu_5_ receptors that are excitatory and induce mobilization of intracellular Ca^2+^ and activation of PKC (Nicoletti et al., 2011).

A number of reports have shown that mGlu_1_ and mGlu_5_ receptors are overexpressed in the spinal cord of ALS patients and SOD1^G93A^ mice (Anneser et al., 1999; Aronica et al., 2001; Brownell et al., 2015; Valerio et al., 2002) and that they contribute to the damage of neuronal and glial cells (D'Antoni et al., 2011; Rossi et al., 2008; Valerio et al., 2002). We found that the mGlu_1_ and mGlu_5_ receptors sited at glutamatergic spinal cord nerve terminals of SOD1^G93A^ mice are abnormally sensitive to the mGlu_1/5_ receptor agonist (*S*)‐3,5‐dihydroxyphenylglycine (3,5‐DHPG), with the mGlu5 receptors being the most involved in this hypersensitivity (Bonifacino, Rebosio, et al., 2019; Giribaldi et al., 2013). Further, we demonstrated that reducing the expression of mGlu_1_ or mGlu_5_ receptors in SOD1^G93A^ mice, overall, ameliorates disease progression (Bonifacino et al., 2017; Bonifacino, Provenzano, et al., 2019; Milanese et al., 2014). Specifically, the genetic ablation of mGlu_5_ receptors produced delayed pathology onset and survival improvement, ameliorated motor skills, enhanced number of preserved MNs, decreased astrocyte and microglia activation, reduced cytosolic free Ca^2+^ and normalized the excessive glutamate release in the spinal cord of SOD1^G93A^ mice (Bonifacino et al., 2017; Bonifacino, Provenzano, et al., 2019).

With this genetically based functional and biochemical background, we have here explored the effects of the pharmacological treatment of SOD1^G93A^ mice with 2‐chloro‐4‐((2,5‐dimethyl‐1‐(4‐(trifluoromethoxy)phenyl)‐1*H*‐imidazol‐4‐yl)ethynyl)pyridine (CTEP), a selective negative allosteric modulator (NAM) of mGlu_5_ receptors. This compound is orally bioavailable and exhibits favourable pharmacokinetic properties (Lindemann et al., 2011). Our results demonstrate that treatment of SOD1^G93A^ mice increased survival probability, reduced progression of the pathology and improved biochemical ALS‐linked outcomes. These effects were more evident in female than in male SOD1^G93A^ mice.

## 2 METHODS

### 2.1 Animals

All animal care and experimental procedures were conducted in accordance with the European Communities Council Directive (EU Directive 114 2010/63/EU for animal experiments; 22 September 2010) and with the Italian D.L. No. 26/2014 and were approved by the local ethics committee and by the Italian Ministry of Health (Project Authorization No. 97/2017‐PR). Animal studies are reported in compliance with the ARRIVE guidelines (Percie du Sert et al., 2020) and with the recommendations made by the British Journal of Pharmacology (Lilley et al., 2020). All efforts were made in following the 3R principles, minimizing animal suffering and using the minimal number of animals necessary to produce statistically reliable results.

B6SJL‐Tg (SOD1*G93A)1Gur mice expressing high copy number of mutant human SOD1 with a Gly93Ala substitution (SOD1^G93A^ mice; Gurney et al., 1994; RRID:MGI:4829804) were originally obtained from The Jackson Laboratory (Bar Harbor, ME, USA). SOD1^G93A^ mice colony was maintained by crossing *SOD1* ^*G93A*^ male mice with background‐matched B6SJL wild‐type (WT; RRID:IMSR_TAC) females and the selective breeding preserved the transgene in the hemizygous state. SOD1^G93A^ mice represent the most widely used animal model for ALS preclinical studies (Kim et al., 2015) because it reproduces several pathological characteristics of ALS in human patients. SOD1^G93A^ develop clear clinical symptoms around 12–13 weeks of life and show the late stage of the disease around 16–18 weeks of life. Mice carrying the SOD1^G93A^ mutation were identified by analysing the tissue extracts from tail tips. Briefly, tissue was homogenized in PBS (Euroclone, Pero, Italy, Cat# ECB4004L), freeze/thawed twice and centrifuged at 23,000× *g* for 15 min at 4°C, and the SOD1 level was evaluated by staining for its enzymic activity on 10% non‐denaturing PAGE by incubation of the gel (45‐min shaking at room temperature) with nitrotetrazolium blue chloride solution (NBT, 1 mg·ml^−1^; Merck, Darmstadt, Germany; Cat# N6876‐5G), followed by incubation (15‐min shaking at room temperature) with riboflavin solution (0.01% w/v; Merck, Darmstadt, Germany; Cat# R4500‐5G) added with tetramethylethylenediamine (TEMED, 0.005% v/v; Carl Roth GmbH Karlsruhe, Germany; Cat# 2367.1).

Both male and female mice were included in the study. Animals were killed by cervical dislocation followed by decapitation (according to the approved protocols of the Italian Ministry of Health; D.L. No. 26/2014, Annex IV, table 3). In survival probability and behavioural experiments, mice were killed at the late stage of the disease (by overdose of isoflurane; according to the approved protocols of the Italian Ministry of Health; D.L. No. 26/2014, Annex IV, table 3), but always before the pathology‐induced death: humane endpoint, according to the clinical score with complete paralysis of hindlimbs (see Table S1), plus righting reflex (Solomon et al., 2011).

### 2.2 Drug treatment

CTEP was synthetized and provided by Prof. Silvana Alfei (Department of Pharmacy, Organic Chemistry Unit, University of Genoa) (Alfei & Baig, 2017). CTEP was suspended in 0.9% NaCl (w/v; HiMedia, Einhausen, Germany, Cat# GRM031) and 0.3% Tween 80 (v/v; Merck, Darmstadt, Germany, Cat# P8074) (Lindemann et al., 2011). Oral suspensions were prepared immediately before their use. SOD1^G93A^ and WT mice were chronically treated with CTEP at 2 mg·kg^−1^ every 48 h or at 4 mg·kg^−1^ every 24 h by using a soft gavage rubber needle (2Biological Instruments, Varese, Italy, Cat# FTP‐20‐38), starting from Day 90 of life (early clinical symptomatic stage) until the end life stage. In histological, pharmacokinetics and release experiments animals were treated for 15 days and killed at Day 105. The dosage was adjusted over time according to the weight of the animals. The orally administered volume was 5 ml·kg^−1^ per mouse, following the guidelines for small animal treatments (Turner et al., 2011). Control age‐matched SOD1^G93A^ and WT mice were treated with the vehicle solution. No signs directly related to the animal handling or to side effects of the drug could be observed.

### 2.3 Behavioural experiments

The effects of the CTEP treatment on disease symptoms were analysed in behavioural tests, three times per week starting at Day 80 of life (10 days before starting the treatment), until killing. Sample sizes and animal numbers were predetermined according to the guidelines for the preclinical in vivo evaluation of pharmacological treatments for ALS/MND (Ludolph et al., 2007). We dedicated four newborn litters for behavioural tasks. The experiments were littermate controlled; the mouse was the experimental unit. The pharmacological treatments were randomized within the cage. The same procedure was applied to histology, pharmacokinetics and release experiments.

We start a first trial with 15 male + 15 female CTEP‐treated (2 mg·kg^−1^ every 48 h) and 15 male + 14 female vehicle‐treated mice. Three female vehicle‐treated mice were not included in the trial (accidental death before Day 100 of life); thus, we concluded the first trial with *n* = 30 SOD1^G93A^ mice (15 males and 15 females) treated with CTEP at the dose of 2 mg·kg^−1^ every 48 h and with *n* = 26 SOD1^G93A^ mice (15 males and 11 females) treated with vehicle. We started the second trial with *n* = 26 SOD1^G93A^ mice (14 males and 12 females) treated with CTEP at the dose of 4 mg·kg^−1^ every 24 h and with *n* = 25 vehicle‐treated SOD1^G93A^ mice (14 males and 11 females). No deaths occurred in this trial; thus, we concluded this second trial with the starting number of animals. The behavioural studies were performed in a randomized order by blinded operators.

#### 2.3.1 Survival probability

Survival time was identified according to the clinical score (see Table S1), plus righting reflex, as the time at which mice were unable to right themselves within 20 s when placed on their side (humane endpoint; Solomon et al., 2011). Animals that reached the humane endpoint were killed as described above.

#### 2.3.2 Body weight

Body weight was measured immediately before behavioural tests as an index of disease progression. Body weight data from male and female vehicle‐ or CTEP‐treated WT animals were used as a benchmark for the graph plots of Figure 2.

#### 2.3.3 Motor coordination

Mice were tested for motor coordination by rotarod and the balance beam tests, starting at Day 80 of life.

##### Rotarod test

The time for which an animal could remain on the rotating cylinder was measured using an accelerating rotarod apparatus (RotaRod 7650; Ugo Basile, Gemonio, VA, Italy; cod: Cat# 47650). In this procedure, the rod rotation gradually increases in speed from 4 to 40 r.p.m. over 5 min. The time of the mouse falling off was recorded. Animals were trained for 1 week before starting experiments.

##### Balance beam test

It consists of 1‐m‐long beam with 6‐mm width upper surface, standing 50 cm above ground. Mice were placed at the starting point and encouraged to cross the beam by means of a black box placed at the end of the beam. The number of foot slips while walking along the beam was recorded (Luong et al., 2011).

#### 2.3.4 Motor function

Mice were rated for motor abilities by scoring the extension reflex of hindlimbs and gait impairment, starting at Day 80 of life.

##### Extension reflex

Animals were evaluated by observing the hindlimb posture when suspended by the tail. Extension reflex score was assigned using a 5‐point score scale (5, no sign of motor dysfunction; 0, complete impairment), as detailed in Table S1, updated from Uccelli et al. (2012).

##### Gait

Deficits were measured by observing the mice freely moving in an open field. Gait score was assigned using a 5‐point score scale (5, no sign of motor dysfunction; 0, complete impairment), as detailed in Table S1, updated from Uccelli et al. (2012).

#### 2.3.5 Muscle strength

Mice were rated for muscle force by using the grip strength (fore limb muscle strength) and the hanging wire (hindlimb paw grip endurance) tests, starting at Day 80 of life.

##### Grip strength

Mice were placed in front of a grasping bar fitted to a force sensor, for the automated detection of the animal forelimb strength. The force transducer has a maximum applicable force of 1500× *g*, with a resolution 0.1 g (GSM Grip‐Strength Meter, Ugo Basile, Gemonio, VA, Italy; Cat# 47200). Animals were suspended by the tail, allowed to grasp the pull bar linked to the motor‐aided dynamometer and pulled by the tail, and the maximal opponent forelimb force was recorded. The test was repeated at least three times per trial, and the average value of the mouse grip force (arbitrary units) was registered excluding outlier values.

##### Hanging wire

Mice were placed on a grid, held approximately 50 cm above a cage containing fresh bedding. The grid was then cautiously turned upside down, and the latency of the animal to release the grid with both hindlimbs was recorded. A 120‐s cut‐off time was applied.

### 2.4 Histological studies

The Immuno‐related procedures used comply with the recommendations made by the *British Journal of Pharmacology*. Animals used on these experiments were not enrolled in behavioural tasks. To minimize the number of animals, the same mouse tissues were used for both MN count and immunofluorescence analysis.

WT or SOD1^G93A^ mice were chronically treated with CTEP 2 mg·kg^−1^ every 48 h (*n* = 12 per group; six males and six females) or vehicle (*n* = 12 per group; six males and six females) for 15 days, by oral gavage. In a second series of experiments, WT or SOD1^G93A^ mice were chronically treated with CTEP 4 mg·kg^−1^ every 24 h (*n* = 12 per group; six males and six females) or vehicle (*n* = 12 per group; six males and six females) for 15 days, by oral gavage.

#### 2.4.1 MN count

CTEP‐ or vehicle‐treated WT and SOD1^G93A^ mice were killed as described above. The spinal cord was dissected at 4°C and postfixed in 4% paraformaldehyde in PBS solution (pH 7.4) for 24 h. After careful rinses in PBS, the specimen was dehydrated with increasing ethanol (VWR, PA, USA, Cat# 20823.293) concentrations (80%, 90%, 95% and 100%) and embedded in Paraplast (Sigma‐Aldrich, St. Louis, MO, USA; Cat# P3558). Transversal Paraplast sections (5 μm thick) were cut, rehydrated in a decreasing ethanol series (100%, 95%, 90% and 80%) and washed in PBS. For MN count, L4/L5 spinal sections were stained using haematoxylin (Merck, Darmstadt, Germany, Cat# H9627) and eosin (Merck, Darmstadt, Germany, Cat# E4382). Microscopic fields of ventrolateral horns were captured with a digital camera coupled to Olympus BX60 microscope, and MNs were counted by light microscopy, on the basis of diameters greater than 25 μm; 2× magnification images were also captured for the identification of cytoplasmic vacuoles in single ventrolateral horn MNs.

#### 2.4.2 Astrogliosis and microgliosis analysis

Dewaxed serial spinal cord sections from CTEP‐ or vehicle‐treated WT and SOD1^G93A^ mice, prepared as described above for MN count, were rehydrated in a decreasing ethanol series (100%, 95%, 90% and 80%) and washed in PBS. Tissues were incubated overnight in a moist chamber at 4°C with a rabbit polyclonal antibody anti‐glial fibrillary acidic protein (GFAP, Sigma‐Aldrich, St. Louis, MO, USA Cat# G9269; RRID:AB477035) or with a rabbit polyclonal antibody anti‐ionized calcium‐binding adapter molecule 1 (IBA‐1, Wako, Osaka, Japan, Cat# 016‐20001; RRID:AB839506). Both antibodies were diluted 1:200 in PBS plus 0.1% BSA (Merck, Darmstadt, Germany, Cat# A9647). After washing with PBS, sections were incubated with Alexa 488‐conjugated anti‐rabbit antiserum secondary antibodies (1:800; Molecular Probes–Thermo Fisher Scientific, Rockford, IL, USA, Cat# A27034; RRID:AB2536097) for 1 h. Sections were then stained with DAPI (Merck, Darmstadt, Germany, Cat# F6057), thoroughly washed in PBS and mounted in a glycerol/PBS (1:1) solution. Sections were examined by an Olympus BX60 epifluorescence microscope. The fluorescence quantification of IBA‐1 or GFAP signals was quantified by the ImageJ free software, Version 1.29 (NIH, Bethesda, MD, USA; RRID:SCR_003070), evaluating the area, the integrated density and the mean grey value of several areas of interest, as experimental replicates. To assess the background, the same analysis has been performed on a non‐fluorescent area. The total fluorescence has been calculated using the following formula: integrated density − (selected area × mean fluorescence of background measures).

### 2.5 Synaptosome preparation and glutamate release experiments

The animals used in these experiments were not enrolled in behavioural tasks. SOD1^G93A^ mice were treated with CTEP (4 mg·kg^−1^ every 24 h, *n* = 6; three males and three females) or vehicle (*n* = 6; three males and three females) for 15 days, starting at Day 90 of life, by oral gavage. WT (*n* = 6; three males and three females), CTEP‐ and vehicle‐treated SOD1^G93A^ mice were killed as described above and the spinal cord was rapidly dissected at 4°C. Spinal cord synaptosomes were prepared essentially as previously described (Raiteri et al., 2007). The tissue was homogenized in 14 vol. of 0.32‐M sucrose (VWR, PA, USA, Cat# 0335) buffered at pH 7.4 with Tris–HCl, using a glass‐Teflon tissue grinder (clearance 0.25 mm; Cat# 432‐0208, Cat# 432‐0202). The homogenate was centrifuged (5 min, 1000× *g* at 4°C) to remove nuclei and debris, and the supernatant was gently stratified on a discontinuous Percoll® (Sigma‐Aldrich, St. Louis, MO, USA; Cat# P1644) gradient (2%, 6%, 10% and 20% v/v in Tris‐buffered sucrose). After centrifugation at 33,500× *g* for 5 min, the layer between 10% and 20% Percoll (synaptosomal fraction) was collected and washed in physiological medium having the following compositions: NaCl, 140 mM; KCl, 3 mM (VWR, PA, USA, Cat# 26764.260); MgSO_4_, 1.2 mM (Merck, Darmstadt, Germany, Cat# 1.05886.1000); NaH_2_PO_4_, 1.2 mM (Merck, Darmstadt, Germany, Cat# 1.06346.0500); NaHCO_3_, 5 mM (Merck, Darmstadt, Germany, Cat# 1.06329.1000); CaCl_2_, 1.2 mM (VWR, PA, USA, Cat# A0222282); HEPES, 10 mM (VWR, PA, USA, Cat# 0511); and glucose, 10 mM (VWR, PA, USA, Cat# 0188) (pH 7.4), centrifuged (20,000× *g* for 15 min at 4°C) and resuspended in physiological medium for release experiments. Synaptosomes purified from WT, CTEP‐ and vehicle‐treated SOD1^G93A^ mice were incubated at 37°C for 15 min in the presence of 0.05‐μM [^3^H]d‐Aspartate ([^3^H]_D_‐Asp), a non‐metabolizable analogue of glutamate used to label the synaptosomal glutamate releasing pools (Fleck et al., 2001; Wang et al., 2007). Aliquots of the synaptosomal suspensions (5‐ to 10‐μg protein) were distributed on microporous filters of nitrocellulose (Merck, Darmstadt, Germany, Cat# DAWP02500) placed at the bottom of a set of parallel superfusion chambers maintained at 37°C (Superfusion System, Ugo Basile, Comerio, VA, Italy; Bonanno & Raiteri, 1987). Superfusion was then started with standard medium at a rate of 0.5 ml·min^−1^. After 36 min of superfusion to equilibrate the system, four 3‐min fractions were collected (*t* = 36–39; 39–42; 42–45; and 45–48 min). 3,5‐DHPG (0.3 or 30 μM) was introduced at the end of the first sample collected (*t* = 39 min) and maintained until the end of the experiment. In the case of KCl depolarization, samples were collected according to the following scheme: two 3‐min samples (*t* = 36–39 and 45–48 min; basal release) before and after one 6‐min sample (*t* = 39–45 min; stimulus‐evoked release). Stimulation with a 90‐s pulse of 15‐mM KCl was applied at *t* = 39 min.

Collected samples and superfused synaptosomes at the end of the experiment were counted for radioactivity by a beta‐counter system (TRI‐CARB 4810TR Liquid Scintillation Analyser; PerkinElmer Holdings Ltd., UK; Cat# A481001). Tritium released in each sample was calculated as fractional rate × 100 (percentage of the total synaptosomal neurotransmitter content at the beginning of the respective collection period). 3,5‐DHPG effects were evaluated by calculating the ratio between the efflux in the fourth sample collected (in which the maximum effect of 3,5‐DHPG was generally reached) and the efflux in the first sample (spontaneous release). This ratio was compared with the corresponding ratio obtained under control condition (in the absence of 3,5‐DHPG). The KCl‐evoked [^3^H]d‐Asp overflow was estimated by subtracting the transmitter content of the two 3‐min samples representing the spontaneous release from the transmitter content in the 6‐min fraction collected during and after the stimulating pulse.

### 2.6 LC–MS analysis of CTEP

WT or SOD1^G93A^ mice were chronically treated with CTEP (4 mg·kg^−1^ every 24 h; *n* = 10 per group; five males and five females) for 15 days, by gavage. Animals used in these experiments were not enrolled in behavioural tasks.

Tissue extracts were prepared by homogenization in ice of the brain or liver in 0.9% NaCl (20% p/v). Samples were added of an equal volume of acetonitrile, vortexed and incubated at 22°C for 60 min. After incubation, samples were centrifuged at 7000× *g* for 10 min, and supernatants were collected. In the case of plasma, obtained by anticoagulation of blood samples with EDTA, 100‐μl sample was added with 100‐μl acetonitrile and then processed as above. CTEP serial dilutions in DMSO and acetonitrile, and tissue brain, liver and plasma from untreated mice were used for calibration curves.

CTEP content was determined by HPLC using a Vanquish (Thermo Fisher Scientific) UHPLC system composed of binary pump, auto‐sampler and column oven. Samples (5 μl) were injected onto a Symmetry 300™ C18 column (150 × 1 mm, 3.5‐μm particle size) maintained at 40°C (Waters Corp., Milford, MA, USA; http://www.waters.com). The eluents were 0.1% formic acid in water (Eluent A) and 0.1% formic acid in acetonitrile (Eluent B). Flow rate was 100 μl·min^−1^, and the elution was 5‐min isocratic 20% B, 15‐min linear gradient to 60% B, 3‐min linear gradient to 100% B, 12‐min isocratic 100% B and 1‐min linear gradient to 20% B. The reequilibration time in 20% B was 14 min. After HPLC separation, the eluent was analysed by Q Exactive Plus Orbitrap mass spectrometer (Thermo Scientific, San Jose, USA) equipped with a heated electrospray ionization source (HESI‐II). Before each acquisition series, the mass spectrometer was calibrated with the positive ion calibration solution (Thermo Fisher Scientific). Samples were analysed by parallel reaction monitoring (PRM) mode under positive polarity condition. Instrument and collision cell (HCD) parameters were optimized by syringe direct infusion of CTEP in acetonitrile. Sheath and auxiliary gas flow rate were 33 and 10, respectively; spray voltage was 4.0 kV; capillary temperature was 250°C; S‐lens RF level was 100; and auxiliary gas heater temperature was 180°C. Software used for operating the UHPLC/HR‐MS was Xcalibur (Version 4.1; RRID:SCR_014593). The PRM mode analysis was conducted at 17,500 resolution. The autogain control (AGC) was optimized at 2 x 10^5^ with a maximum injection time (maxIT) of 250 ms, and the normalized collision energy (NCE) for fragmentation of CTEP was optimized at 95. Quantitation of CTEP in PRM mode was based on fragmentation of precursor ion 392 *m*/*z* (CTEP) to 128.05 *m*/*z* production. CTEP final concentration was then expressed as μg·g^−1^ brain tissue or μg·ml^−1^ plasma.

### 2.7 Data and statistical analysis

The data and statistical analysis comply with the recommendations of the *British Journal of Pharmacology* on experimental design and analysis in pharmacology (Curtis et al., 2015). All the experimental groups were designed using a randomization protocol (https://www.graphpad.com/quickcalcs/randomize1/). Statistical analysis was undertaken only for studies where each group size was at least *n* = 5. Data for statistical analysis based on individual mouse replicates were not used as independent values. Normal distribution was determined by Shapiro–Wilk test (http://www.statskingdom.com/320ShapiroWilk.html) and outliers by Grubbs' test (https://www.graphpad.com/quickcalcs/Grubbs1.cfm). The Kaplan–Meier analysis was applied to evaluate survival probability, and cumulative curves were compared using the log‐rank test. Statistical comparison of two means was performed by unpaired two‐tailed Student's *t* test. Multiple comparisons were performed using the ANOVA (one‐ and two‐way ANOVA) followed by Bonferroni's or Tukey's post hoc test. The post hoc tests were performed only in the presence of an overall statistically significant difference in group means. The threshold for statistical significance (*p*) was set at *p* < .05. Data are always presented as mean ± SEM.

The statistical analysis of in vivo studies using the low dose of CTEP was performed utilizing 30 (15 males and 15 females) 2‐mg·kg^−1^ every 48 h CTEP‐treated SOD1^G93A^ mice versus 26 (15 males and 11 females) vehicle‐treated SOD1^G93A^ mice and 10 (five males and five females) 2‐mg·kg^−1^ every 48 h CTEP‐treated WT mice versus 10 (five males and five females) vehicle‐treated WT mice. The statistical analysis of in vivo studies using the high dose of CTEP was performed utilizing 25 (14 males and 12 females) 4 mg·kg^−1^ every 24 h CTEP‐treated SOD1^G93A^ mice versus 25 (14 males and 11 females) vehicle‐treated SOD1^G93A^ mice and 10 (five males and five females) 4‐mg·kg^−1^ every 24 h CTEP‐treated WT mice versus 10 (five males and five females) vehicle‐treated WT mice. Histochemistry statistics was performed using *n* = 12 (six males and six females) mice per group (2‐mg·kg^−1^ every 48 h CTEP‐treated SOD1^G93A^ or WT mice and vehicle‐treated SOD1^G93A^ or WT mice; 4‐mg·kg^−1^ every 24 h CTEP‐treated SOD1^G93A^ or WT mice and vehicle‐treated SOD1^G93A^ or WT mice) for a total of 48 mice. Neurotransmitter release statistics was performed with a gender‐balanced number of *n* = 6 mice (three males and three females) per group (4‐mg·kg^−1^ every 24 h CTEP‐treated SOD1^G93A^ or vehicle‐treated SOD1^G93A^ or WT mice) for a total of 18 mice. Statistical analysis for CTEP distribution in plasma and tissues was performed using *n* = 10 (five males and five females) mice per group (4 mg·kg^−1^ every 24 h CTEP‐treated SOD1^G93A^ or WT mice) for a total of 20 mice. The SigmaStat Software (for Windows Version 3.5‐2006, Registration Number 773050002, RRID:SCR_010285, Inc., San Jose, CA, USA) was used for statistical analysis and the SigmaPlot Software (for Windows, Version 10.0‐2006, Registration Number 775060002, RRID:SCR_003210, Inc., San Jose, CA, USA) for figure plots.

### 2.8 Materials

3,5‐DHPG was supplied by Tocris (Cat# 0805; Bristol, UK) and [^3^H]d‐Asp by PerkinElmer Holdings Ltd. (Cat# NET581001MC; Waltham, Massachusetts, USA).

### 2.9 Nomenclature of targets and ligands

Key protein targets and ligands in this article are hyperlinked to corresponding entries in http://www.guidetopharmacology.org and are permanently archived in the Concise Guide to PHARMACOLOGY 2019/20 (Alexander, Christopoulos et al., 2019; Alexander, Kelly et al., 2019; Alexander, Mathie et al., 2019).

## 3 RESULTS

### 3.1 Survival probability and weight loss in CTEP‐treated SOD1^G93A^ mice

**FIGURE 1:**
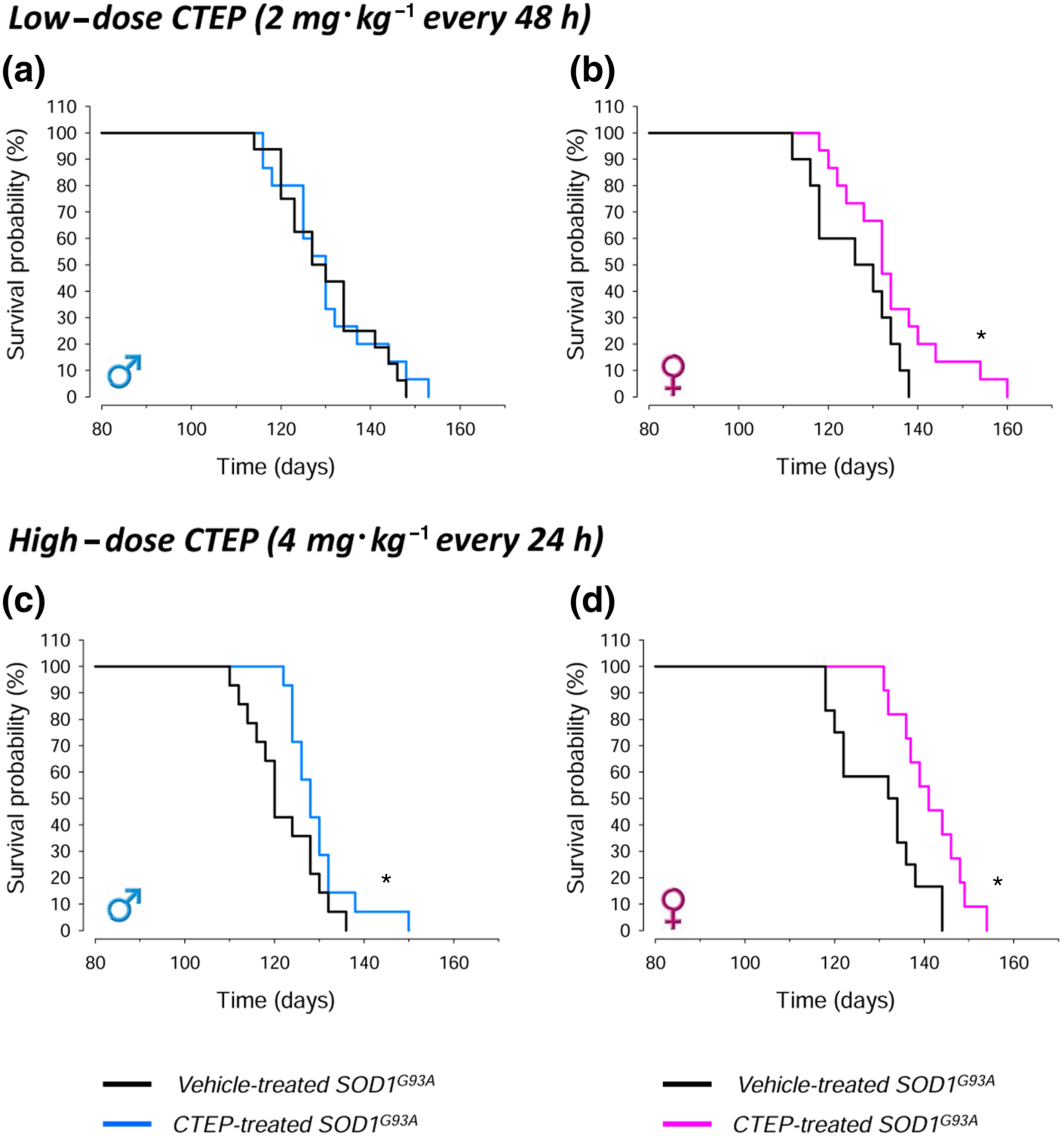
Survival probability in CTEP‐ and vehicle‐treated SOD1^G93A^ mice. Survival was measured in CTEP‐ or vehicle‐treated male and female mice, at low‐dose (2 mg·kg^−1^ every 48 h) or high‐dose (4 mg·kg^−1^ per 24 h) CTEP. CTEP was orally administered by oral gavage starting at Day 90 of age and maintained until the animals were killed. Survival time was determined as the time when the animals was unable to right itself within 20 s when placed on their side. Data in (a) were obtained from 15 low‐dose CTEP‐treated and 15 vehicle‐treated SOD1^G93A^ male mice. Data in (b) were obtained from 15 low‐dose CTEP‐treated and 11 vehicle‐treated SOD1^G93A^ female mice. Data in (c) were obtained from 14 high‐dose CTEP‐treated and 14 vehicle‐treated SOD1^G93A^ male mice. Data in (d) were obtained from 12 high‐dose CTEP‐treated and 11 vehicle‐treated SOD1^G93A^ female mice. In (b)–(d), *P < 0.05, significantly different from vehicle‐treated SOD1^G93A^ mice; Kaplan–Meier analysis with log‐rank test

Survival and body weight were monitored in SOD1^G93A^ mice treated with low‐dose (2 mg·kg^−1^ every 48 h) or high‐dose (4 mg·kg^−1^ every 24 h) CTEP, compared with gender‐ and age‐matched vehicle‐treated mice (Figure 1). The same animals were tested for the behavioural analyses reported below.

After treatment with low‐dose CTEP, no significant survival improvement was observed in CTEP‐treated male mice (Figure 1a), whereas a significant increase was present in CTEP‐treated female mice (Figure 1b). Based on this encouraging trend, we designed a second trial with an higher dose of CTEP (4 mg·kg^−1^ every 24 h). In contrast to the low‐dose CTEP trial, high‐dose CTEP significantly prolonged survival in SOD1^G93A^ males (Figure 1c) and produced a further increase of lifespan in females (Figure 1d). The average median survival values are reported in Table S2.

**FIGURE 2:**
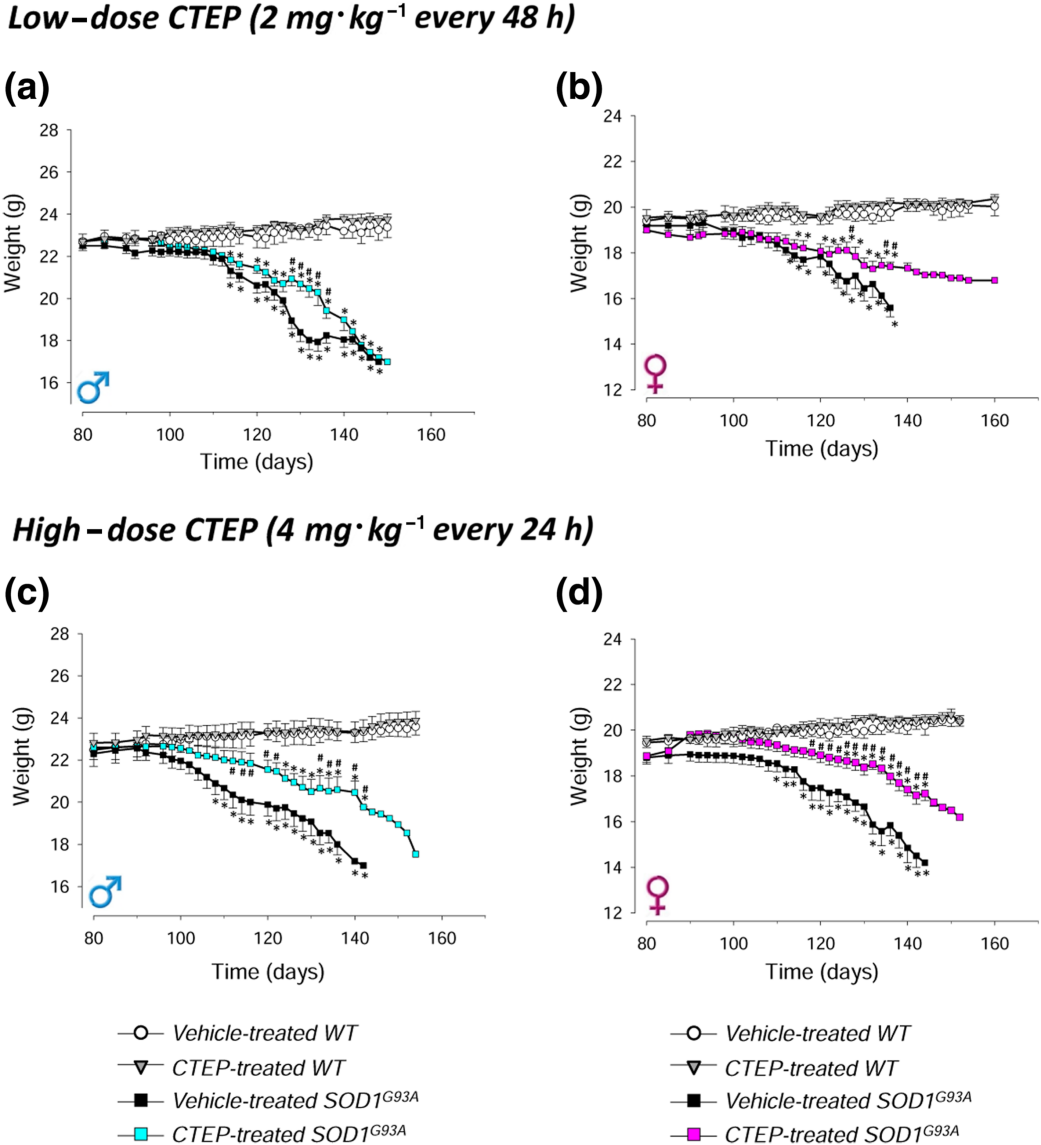
Body weight in CTEP‐ and vehicle‐treated SOD1^G93A^ and WT mice. The body weight loss was measured as an index of disease progression in CTEP‐ and vehicle‐treated SOD1^G93A^ or WT male and female mice, at low‐dose (2 mg·kg^−1^ every 48 h) or high‐dose (4 mg·kg^−1^ every 24 h) CTEP. Animals were weighed 3 days a week starting on Day 80 of life. Data in (a) are means ± SEM of 15 low‐dose CTEP‐treated and 15 vehicle‐treated SOD1^G93A^ male mice and of five low‐dose CTEP‐treated and five vehicle‐treated WT male mice. Data in (b) are means ± SEM of 15 low‐dose CTEP‐treated and 11 vehicle‐treated SOD1^G93A^ female mice and of five low‐dose CTEP‐treated and five vehicle‐treated WT female mice. Data in (c) are means ± SEM of 14 high‐dose CTEP‐treated and 14 vehicle‐treated SOD1^G93A^ male mice and five high‐dose CTEP‐treated and five vehicle‐treated WT male mice. Data in (d) are means ± SEM of 12 high‐dose CTEP‐treated and 11 vehicle‐treated SOD1^G93A^ female mice and five high‐dose CTEP‐treated and five vehicle‐treated WT female mice. *P < 0.05, significantly different from CTEP‐ or vehicle‐treated WT mice, respectively; ^#^ *P* < 0.05, significantly different from vehicle‐treated SOD1^G93A^ mice; two‐way ANOVA followed by Tukey's post hoc test

The body weight loss, an index of the disease progression, was studied in CTEP‐ and vehicle‐treated SOD1^G93A^ and WT male and female mice. WT mice body weight was almost stable during the examination period, and no variations due to low‐ or high‐dose CTEP treatment were observed (Figure 2a–d). Both CTEP‐ and vehicle‐treated SOD1^G93A^ mice showed a significant decrease of body weight, compared with gender‐ and treatment‐matched WT mice, but the weight loss was always less pronounced in the CTEP‐treated groups. In the high‐dose CTEP trial, weight loss of SOD1^G93A^ males (Figure 2c) and females (Figure 2d) was less pronounced with respect to that of males (Figure 2a) and females (Figure 2b) treated with the low‐dose CTEP. Furthermore, the drug treatment produced a delay of body weight loss with respect to vehicle‐treated sex‐matched groups.

### 3.2 Motor coordination skills in CTEP‐treated SOD1^G93A^ mice

**FIGURE 3:**
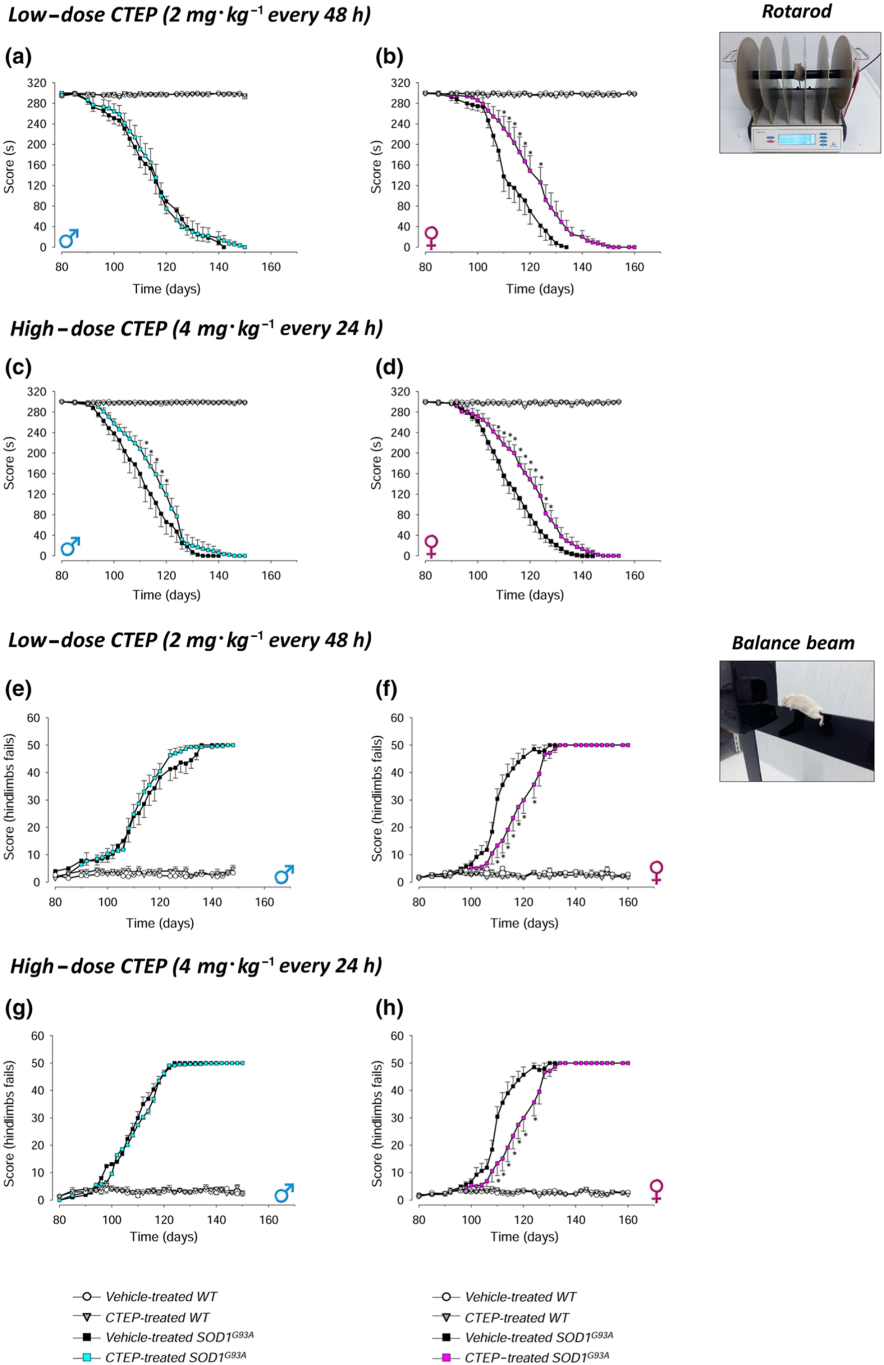
Rotarod and balance beam tests in CTEP‐ and vehicle‐treated SOD1^G93A^ and WT mice. The rotarod (a–d) and balance beam (e–h) tests were performed during the disease progression in CTEP‐ and vehicle‐treated SOD1^G93A^ or WT male and female mice, at low‐dose (2 mg·kg^−1^ every 48 h) or high‐dose (4 mg·kg^−1^ every 24 h) CTEP. Animals were tested 3 days a week starting on Day 80 of life. The rod speed of the rotarod was increased from 4 to 40 r.p.m. in 5 min, and falling off time was recorded. The number of hindlimbs fails during the beam crossing was registered in the balance beam test. Data in (a) and (e) are means ± SEM of 15 low‐dose CTEP‐treated and 15 vehicle‐treated SOD1^G93A^ male mice and of five low‐dose CTEP‐treated and five vehicle‐treated WT male mice. Data in (b) and (f) are means ± SEM of 15 low‐dose CTEP‐treated and 11 vehicle‐treated SOD1^G93A^ female mice and of five low‐dose CTEP‐treated and five vehicle‐treated WT female mice. Data in (c) and (g) are means ± SEM of 14 high‐dose CTEP‐treated and 14 vehicle‐treated SOD1^G93A^ male mice and of five high‐dose CTEP‐treated and five vehicle‐treated WT male mice. Data in (d) and (h) are means ± SEM of 12 high‐dose CTEP‐treated and 11 vehicle‐treated SOD1^G93A^ female mice and of five high‐dose CTEP‐treated and five vehicle‐treated WT female mice. *P < 0.05, significantly different from vehicle‐treated SOD1^G93A^ mice; two‐way ANOVA followed by Tukey's post hoc test

Motor coordination was monitored during disease progression by rotarod and balance beam tasks in male and female WT or SOD1^G93A^ mice treated with low‐dose (2 mg·kg^−1^ every 48 h) or high‐dose (4 mg·kg^−1^ every 24 h) CTEP, compared with gender‐ and age‐matched vehicle‐treated WT or SOD1^G93A^ mice (Figure 3).

As shown in the Figure 3a–h, WT vehicle‐ or CTEP‐treated mice performed maximally during the entire observation period. Moreover, low‐ or high‐dose CTEP did not modify WT mouse performance (rotarod [Figure 3a–d] or balance beam [Figure 3e–h]). The rotarod performances of low‐dose CTEP‐ and vehicle‐treated SOD1^G93A^ mice were superimposable in males (Figure 3a), whereas a significant increase was observed at the high‐dose CTEP (Figure 3c). Differently from males, CTEP‐treated females showed a significant amelioration both at low‐dose (Figure 3b) and at high‐dose (Figure 3d) CTEP. In the balance beam test, no modifications were observed in males at low‐dose CTEP (Figure 3e) or high‐dose CTEP (Figure 3g). In females, both CTEP doses produced a marked, statistically significant, improvement of the balance beam test performances (Figure 3f,h).

### 3.3 Motor function skills in CTEP‐treated SOD1^G93A^ mice

**FIGURE 4:**
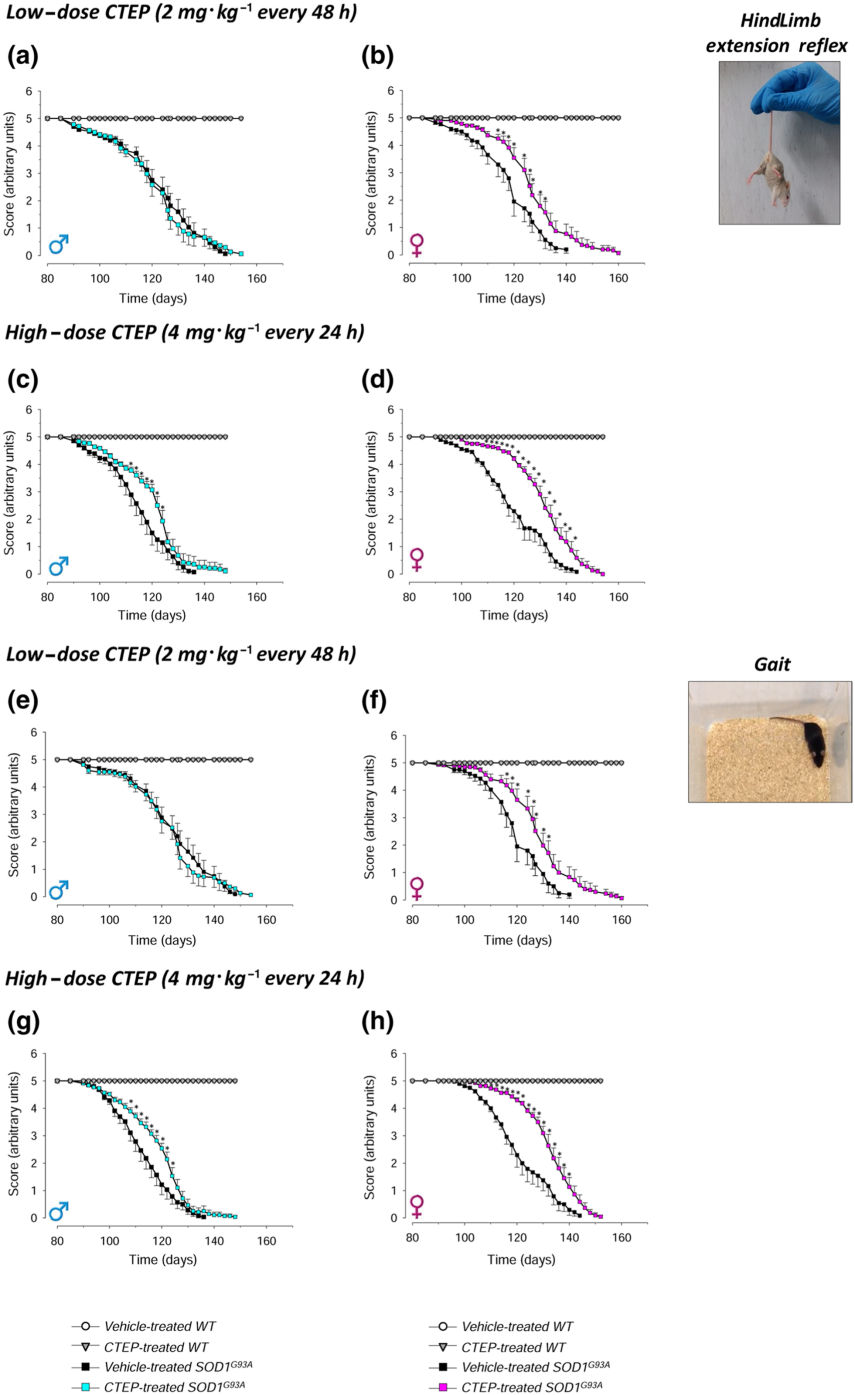
Hindlimb extension reflex and gait impairment tests in CTEP‐ and vehicle‐treated SOD1^G93A^ and WT mice. The hindlimb extension reflex (a–d) and gait impairment (e–h) tests were performed during the disease progression in CTEP‐ and vehicle‐treated SOD1^G93A^ or WT male and female mice, at low‐dose (2 mg·kg^−1^ every 48 h) or high‐dose (4 mg·kg^−1^ every 24 h) CTEP. Animals were tested 3 days a week starting on Day 80 of life. Animals were suspended by the tail, and a 0–5 scale was used to score hindlimb posture. To evaluate gait impairment, mice were allowed to move in an open field, and a 0–5 scale score was used. Data in (a) and (e) are means ± SEM of 15 low‐dose CTEP‐treated and 15 vehicle‐treated SOD1^G93A^ male mice and of five low‐dose CTEP‐treated and five vehicle‐treated WT male mice. Data in (b) and (f) are means ± SEM of 15 low‐dose CTEP‐treated and 11 vehicle‐treated SOD1^G93A^ female mice and of five low‐dose CTEP‐treated and five vehicle‐treated WT female mice. Data in (c) and (g) are means ± SEM of 14 high‐dose CTEP‐treated and 14 vehicle‐treated SOD1^G93A^ male mice and of five high‐dose CTEP‐treated and five vehicle‐treated WT male mice. Data in (d) and (h) are means ± SEM of 12 high‐dose CTEP‐treated and 11 vehicle‐treated SOD1^G93A^ female mice and of five high‐dose CTEP‐treated and five vehicle‐treated WT female mice. *P < 0.05, significantly different from vehicle‐treated SOD1^G93A^ mice; two‐way ANOVA followed by Tukey's post hoc test

We monitored extension reflex of hindlimbs and gait impairment in WT or SOD1^G93A^ mice treated with low‐dose (2 mg·kg^−1^ every 48 h) or high‐dose (4 mg·kg^−1^ every 24 h) CTEP, compared with gender‐ and age‐matched vehicle‐treated WT or SOD1^G93A^ mice (Figure 4), as an index of motor abilities.

The performance of vehicle‐treated male and female WT mice did not decrease during time and CTEP did not modify WT mouse extension reflex (Figure 4a–d) or gait (Figure 4e–h). Low‐dose CTEP‐treated male SOD1^G93A^ mice did not show improvement of extension reflex score compared with vehicle‐treated SOD1^G93A^ mice (Figure 4a), whereas the treatment with high‐dose CTEP produced a significant amelioration (Figure 4c). Instead, low‐dose CTEP‐treated SOD1^G93A^ female mice exhibited a significant amelioration of this task (Figure 4b), which was further enhanced at high‐dose CTEP (Figure 4d). A similar pattern was observed in gait test: low‐dose CTEP‐treated SOD1^G93A^ male mice did not perform better than vehicle‐treated mice (Figure 4e), whereas the treatment with the high dose produced a significant amelioration of the score (Figure 4g). CTEP‐treated SOD1^G93A^ female mice exhibited a significant amelioration both at the low and high doses of CTEP (Figure 4f,h), being the effect more pronounced at high‐dose CTEP.

### 3.4 Hindlimb and forelimb muscle strength in CTEP‐treated SOD1^G93A^ mice

**FIGURE 5:**
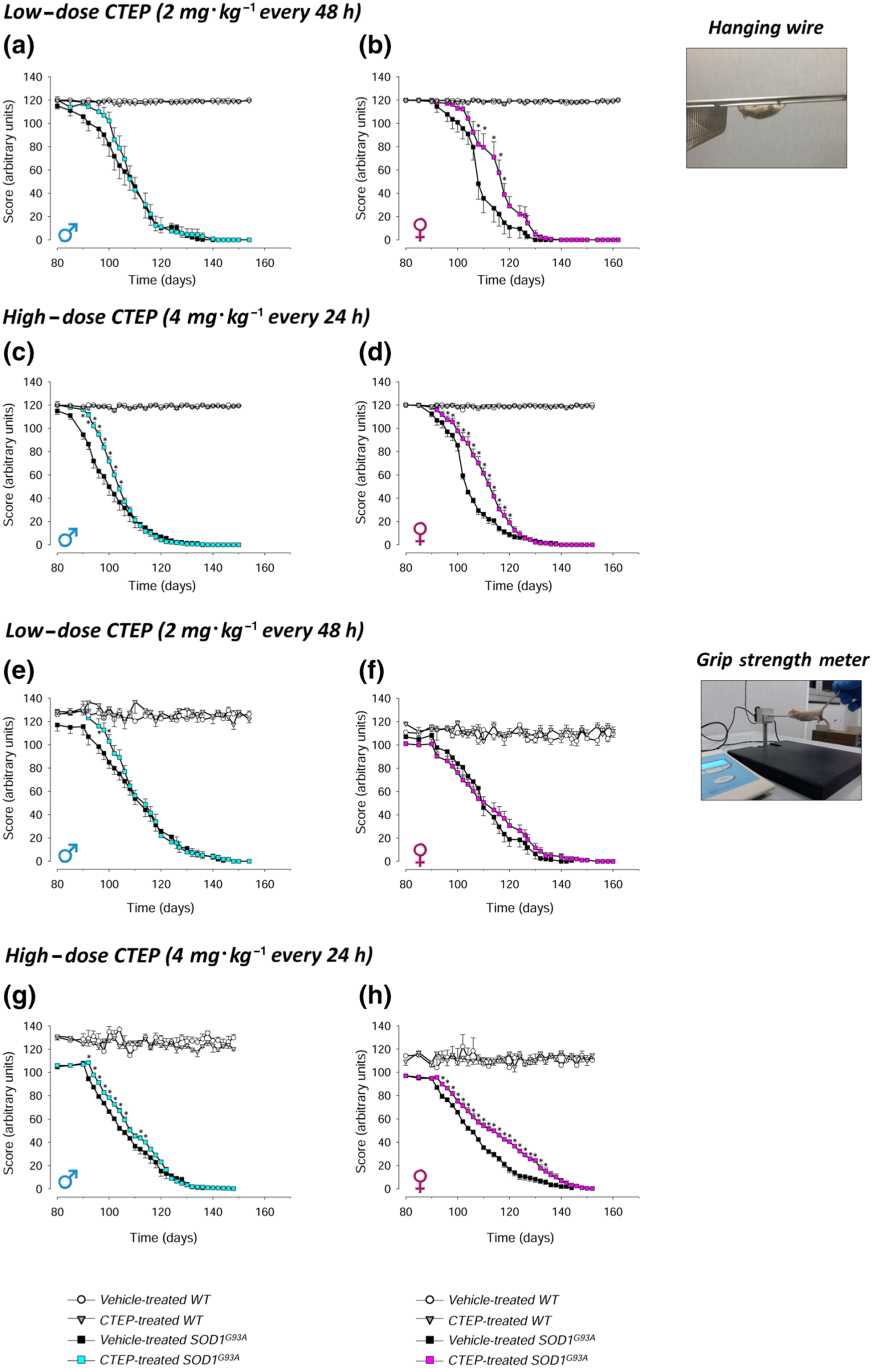
Hanging wire and grip strength meter tests in vehicle‐ and CTEP‐treated SOD1^G93A^ and WT mice. The hanging wire (a–d) and grip strength meter (e–h) tests were performed during the disease progression in CTEP‐ and vehicle‐treated SOD1^G93A^ or WT male and female mice, at low‐dose (2 mg·kg^−1^ every 48 h) or high‐dose (4 mg·kg^−1^ every 24 h) CTEP. Animals were tested 3 days a week starting on Day 80 of life. In the hanging wire test, animals were placed on a grid that was gently turned upside down, and the hindlimb detach time was recorded. In the grip strength metre test, animals were allowed to grasp the pull bar of a motor‐aided dynamometer and pulled by the tail; the maximal opponent forelimbs force was recorded. Data in (a) and (e) are means ± SEM of 15 low‐dose CTEP‐treated and 15 vehicle‐treated SOD1^G93A^ male mice and of five low‐dose CTEP‐treated and five vehicle‐treated WT male mice. Data in (b) and (f) are means ± SEM of 15 low‐dose CTEP‐treated and 11 vehicle‐treated SOD1^G93A^ female mice and of five low‐dose CTEP‐treated and five vehicle‐treated WT female mice. Data in (c) and (g) are means ± SEM of 14 high‐dose CTEP‐treated and 14 vehicle‐treated SOD1^G93A^ male mice and of five high‐dose CTEP‐treated and five vehicle‐treated WT male mice. Data in (d) and (h) are means ± SEM of 12 high‐dose CTEP‐treated and 11 vehicle‐treated SOD1^G93A^ female mice and of five high‐dose CTEP‐treated and five vehicle‐treated WT female mice. *P < 0.05, significantly different from vehicle‐treated SOD1^G93A^ mice; two‐way ANOVA followed by Tukey's post hoc test

We monitored the hindlimb and forelimb muscle strength by the hanging wire and the grip strength meter test, respectively, in WT or SOD1^G93A^ mice treated with low‐dose (2 mg·kg^−1^ every 48 h) or high‐dose (4 mg·kg^−1^ every 24 h) CTEP compared with the gender‐ and age‐matched vehicle‐treated WT or SOD1^G93A^ mice (Figure 5).

No variations were observed in vehicle‐treated and low‐ or high‐dose CTEP‐treated WT male and female performances in the hanging wire (Figure 5a–d) or grip strength (Figure 5e–h) test values. Treatment with the low‐dose CTEP induced a non‐significant amelioration of the hanging wire test score in SOD1^G93A^ male mice only in the first period of disease progression (Figure 5a). This effect became significant with high‐dose CTEP (Figure 5c). Female mice showed a significant improvement at both the low‐ and high‐dose CTEP (Figure 5b,d). As to the grip strength meter, low‐dose CTEP produced a small but significant improvement in male mice in the first phase of treatment (Figure 5e), which became more evident at the high dose (Figure 5g). The performances of low‐dose CTEP‐treated SOD1^G93A^ females did not differ from those of vehicle‐treated mice, whereas a significant amelioration was produced by high‐dose CTEP.

### 3.5 Spinal MN loss, astrogliosis and microgliosis in CTEP‐treated SOD1^G93A^ mice

**FIGURE 6:**
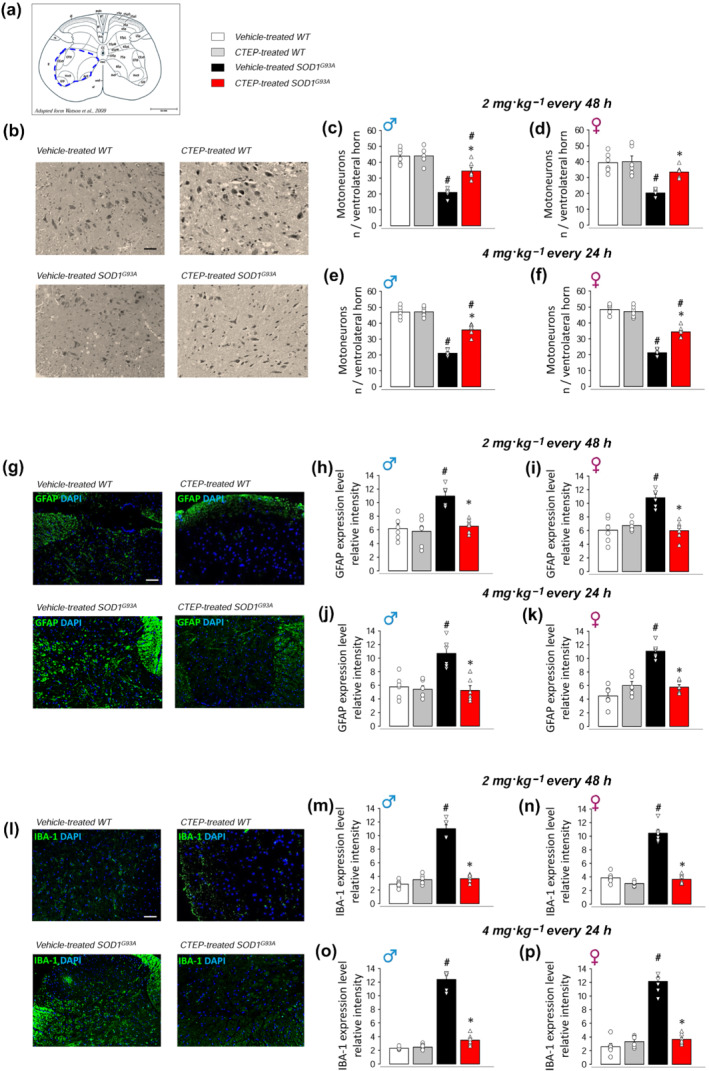
Motor neuron loss, astrogliosis and microgliosis in the spinal cord of vehicle‐ and CTEP‐treated SOD1^G93A^ and WT mice. Motor neuron (MN) loss, astrogliosis and microgliosis have been assessed in ventrolateral horn of L4–L5 spinal cord sections (5 μm thick; reference area of analysis in a; adapted from Watson et al., 2009) from CTEP‐ and vehicle‐treated SOD1^G93A^ or WT mice, at low‐dose (2 mg·kg^−1^ every 48 h) or high‐dose (4 mg·kg^−1^ every 24 h) CTEP. MNs were counted after staining with haematoxylin and eosin and selected on the basis of diameters >25 μm. (b) Representative light microscopy photomicrographs (scale bar 100 μm) from high‐dose CTEP‐ and vehicle‐treated SOD1^G93A^ and WT male mice. Quantification of MN number in low‐dose CTEP‐ and vehicle‐treated SOD1^G93A^ and WT male (c) and female (d) mice and high‐dose CTEP‐ and vehicle‐treated SOD1^G93A^ and WT male (e) and female (f) mice is reported. The expression of GFAP was measured as an index of astrogliosis by immunofluorescence (IF) staining and epifluorescence microscopy acquisition using mouse anti‐GFAP monoclonal antibody and DAPI staining for nuclei. (g) Representative IF images (scale bar 100 μm) from high‐dose CTEP‐ and vehicle‐treated SOD1^G93A^ and WT male mice. Quantification of GFAP IF intensity in low‐dose CTEP‐ and vehicle‐treated SOD1^G93A^ and WT male (h) and female (i) mice and in high‐dose CTEP‐ and vehicle‐treated SOD1^G93A^ and WT male (l) and female (m) mice is reported. The expression of IBA‐1 was measured as an index of microglia activation by IF staining and epifluorescence microscopy acquisition using goat anti‐IBA‐1 polyclonal antibody and DAPI staining for nuclei. (n) Representative IF images (scale bar 100 μm) from high‐dose CTEP‐ and vehicle‐treated SOD1^G93A^ and WT male mice. Quantification of IBA‐1 IF intensity in low‐dose CTEP‐ and vehicle‐treated SOD1^G93A^ and WT male (o) and female (p) mice and in high‐dose CTEP‐ and vehicle‐treated SOD1^G93A^ and WT male (q) and female (r) mice is reported. Data are means ± SEM of six biological replicates (*n* = 6 mice per group per sex). Scatter plot represents single biological replicates. *P < 0.05, significantly different from vehicle‐treated SOD1^G93A^; ^#^ *P* < 0.05, significantly different from corresponding WT mice; two‐way ANOVA followed by Bonferroni's post hoc test

We performed histological analyses in the ventrolateral horns of L4–L5 lumbar spinal cord to assess MN loss and astrocyte or microglia activation state in male and female WT or SOD1^G93A^ mice treated with the low (2 mg·kg^−1^ every 48 h) or high (4 mg·kg^−1^ every 24 h) dose of CTEP, compared with gender‐ and age‐matched vehicle‐treated WT or SOD1^G93A^ mice (Figure 6).

Figure 6a shows the ventral horn area of the spinal cord investigated in these experiments. Figure 6b shows representative histological images from WT and SOD1^G93A^ male mice treated with vehicle or 4 mg·kg^−1^ every 24 h CTEP. Vehicle‐treated SOD1^G93A^ mice showed tissue damage associated with severe neuronal loss when compared with WT mice, whereas CTEP‐treated mice displayed improved histological features and significant MN preservation. The quantification of the number of MNs is reported in Figure 6c–f. MNs were significantly reduced in vehicle‐treated SOD1^G93A^ with respect to WT mice, and CTEP treatment significantly prevented this reduction. Unexpectedly, no differences of CTEP effects were found regardless of low vs. high doses and sex. Spinal SOD1^G93A^ MNs contain numerous cytoplasmic vacuoles, even at the early stage of the disease (Vinsant et al., 2013). Our histological analyses confirmed the presence of vacuoles in MNs from vehicle‐treated SOD1^G93A^ mice, while show a marked reduction in high‐dose CTEP‐treated mice (see Figure S1).

Figure 6g shows representative epifluorescence microscopy images of GFAP expression in vehicle‐treated and CTEP‐treated (4 mg·kg^−1^ every 24 h) WT and SOD1^G93A^ mice. The quantitative analysis indicated that the expression of GFAP was strongly increased in vehicle‐treated SOD1^G93A^ with respect to WT mice and that GFAP overexpression was abolished in CTEP‐treated SOD1^G93A^ mice, independently from the dose and sex (Figure 6h–k). Figure 6l shows representative epifluorescence microscopy images of IBA‐1 expression. Expression of IBA‐1 was markedly higher in vehicle‐treated SOD1^G93A^ with respect to WT mice, and IBA‐1 overexpression was inhibited in CTEP‐treated SOD1^G93A^ mice (Figure 6m–p). Again, effects were comparable between low‐ and high‐dose CTEP and in males vs. females.

### 3.6 Glutamate release in the spinal cord of CTEP‐treated SOD1^G93A^ mice

**FIGURE 7:**
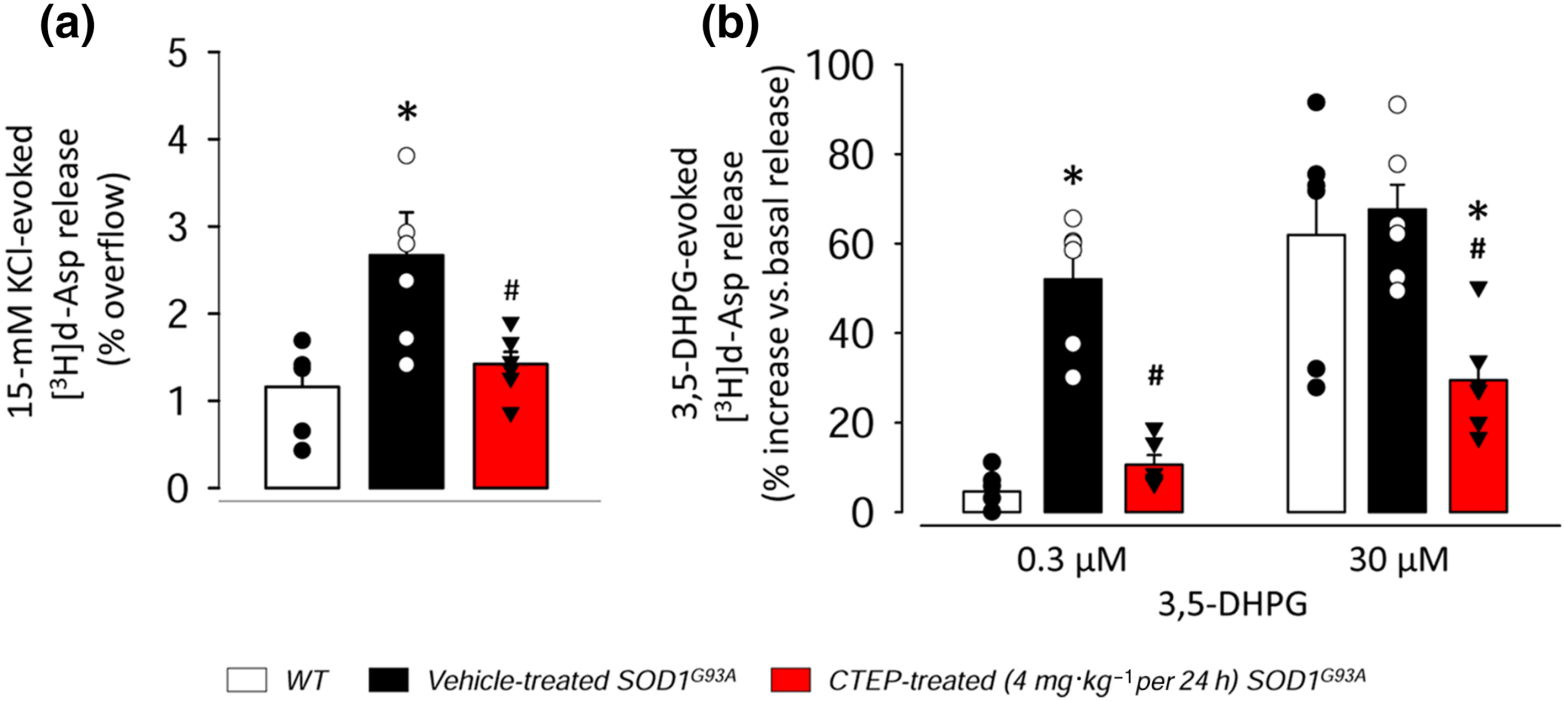
[^3^H]d‐Aspartate release from spinal cord synaptosomes of WT, vehicle‐ and CTEP‐treated SOD1^G93A^ mice. Neurotransmitter release was measured in spinal cord synaptosomes purified from gender‐balanced vehicle‐, CTEP‐treated (4 mg·kg^−1^ every 24 h) and aged‐matched WT mice. (a) 15‐mM KCl‐evoked [^3^H]d‐Asp and (b) 3,5‐DHPG‐induced [^3^H]d‐Asp release are reported. Synaptosomes were loaded with 0.05 μM of [^3^H]d‐Asp, to label the glutamatergic intraterminal releasing pools, and exposed, in superfusion to 15‐mM KCl and 0.3‐ or 30‐μM 3,5‐DHPG. Superfusion samples were quantified for radioactivity. Results are expressed as % of radioactivity released by 15‐mM KCl (% overflow; a) or % increase of basal release induced by 3,5‐DHPG (b). Data are expressed as means ± SEM of six independent experiments run in triplicate (*n* = 6 mice per group, three males and three females; three superfusion chambers per mouse for each single experiment). Scatter plot represents single biological replicate. *P < 0.05, significantly different from WT mice; ^#^ *P* < 0.05, significantly different from vehicle‐treated SOD1^G93A^ mice; one‐way ANOVA followed by Bonferroni's post hoc test

Abnormal depolarization‐evoked (Milanese et al., 2011) and mGlu1/5 receptor‐induced (Giribaldi et al., 2013) release of glutamate takes place in the spinal cord of SOD1^G93A^ mice. We investigated here whether chronic treatment with CTEP (4 mg·kg^−1^ every 24 h) could affect the abnormal glutamate release in sex‐balanced SOD1^G93A^ mice (Figure 7). The depolarization‐evoked exocytotic release of [^3^H]d‐Asp from spinal cord synaptosomes was increased almost threefold in vehicle‐treated SOD1^G93A^ mice with respect to WT mice. CTEP treatment abolished this excessive [^3^H]d‐Asp release (Figure 7a). The mGlu_1/5_ receptor agonist 3,5‐DHPG, applied at 0.3 μM, induced [^3^H]d‐Asp release in SOD1^G93A^ mice but not in WT mice. The 0.3 μM 3,5‐DHPG‐induced [^3^H]d‐Asp release was abolished in spinal cord synaptosomes purified from CTEP‐treated SOD1^G93A^ mice. The 30‐μM 3,5‐DHPG‐induced [^3^H]d‐Asp release in SOD1^G93A^ mice was comparable to that in WT mice, as previously reported (Giribaldi et al., 2013; Figure 7b). Also the 30‐μM 3,5‐DHPG‐induced [^3^H]d‐Asp release was significantly reduced by in vivo CTEP treatment.

### 3.7 CTEP distribution in the brain, liver and plasma

To investigate whether the clinical differences observed in the behaviour experiments might be due to a different distribution and access of CTEP to the brain, we treated WT or SOD1^G93A^ male and female mice with CTEP (4 mg·kg^−1^ every 24 h) and measured the CTEP content in extracts of the brain by MS. The CTEP content was also measured in the liver and plasma of the same animals.

The analysis of CTEP distribution in the different compartments showed no significant differences in sex‐matched SOD1^G93A^ mice in the brain, liver and plasma (Table 1). No differences were also observed between male and female WT mice, except for the CTEP content in plasma.

**TABLE 1:** CTEP distribution in WT and SOD1^G93A^ mice

	SOD1^G93A^	
	Male	Female
Brain (μg·g^−1^)	7.93 ± 2.96, *n* = 5	8.36 ± 1.32, *n* = 5
Liver (μg·g^−1^)	3.40 ± 1.25, *n* = 5	4.22 ± 1.39, *n* = 5
Plasma (μg·ml^−1^)	1.22 ± 0.13, *n* = 5	0.83 ± 0.11, *n* = 5

	WT	
	Male	Female
Brain (μg·g^−1^)	4.07 ± 1.41, *n* = 5	5.76 ± 2.57, *n* = 5
Liver (μg·g^−1^)	2.54 ± 0.98, *n* = 5	4.47 ± 1.12, *n* = 5
Plasma (μg·ml^−1^)	0.69 ± 0.14, *n* = 5*	1.37 ± 0.23, *n* = 5

*Note:* CTEP concentration in the brain (μg·g^−1^), liver (μg·g^−1^) and plasma (μg·ml^−1^) after 15‐day CTEP administration (4 mg·kg^−1^ every 24 h) starting at Day 90 in SOD1^G93A^ and age‐matched WT mice. Data are mean ± SEM of five independent analyses.

* *P* < 0.05, significantly different from female WT mice; two‐way ANOVA followed by Bonferroni's post hoc test.

## 4 DISCUSSION

Despite important scientific progress, ALS still represents a neurodegenerative disease without clinically effective therapy. At present, only two drugs, riluzole and edaravone, have been approved for ALS patients. The former decreases glutamatergic transmission mainly blocking voltage‐gated sodium channels (Hebert et al., 1994) and also reduces Ca^2+^ recruitment through direct activation of G proteins and by preventing NMDA receptor activation (Doble, 1996). Unfortunately, it has a very unspecific mechanism of action and it increases patient survival by only a few months, with no evidence for amelioration of the quality of life (Lu et al., 2016). Edaravone has been approved by Food and Drug Administration (FDA) in 2017, it acts as free radical scavenger and prevents oxidative stress damage to neurons. It is efficacious in early stage ALS patients only (Abe et al., 2017). Therefore, there is an urgent need to translate the current scientific knowledge into effective therapies.

In the complex scenario of ALS, glutamate transmission and the downstream excitotoxic events have been long proposed as a pathological charcteristic of both fALS and sALS and still represent a widely studied issue (King et al., 2016; Van Den Bosch et al., 2006). In this framework, we and others focussed on the role of mGlu_5_ receptors in ALS progression. These receptors are actively involved in many pathophysiological cellular processes, such as maintenance of glutamate homeostasis and synaptic plasticity (Panatier & Robitaille, 2016; Vermeiren et al., 2005), glial activation (Aronica et al., 2000; D'Antoni et al., 2008), mitochondria dysfunction and oxidative stress (Li et al., 2011), intracellular calcium levels and downstream signalling cascades (Liu et al., 2014), neuroinflammation (Drouin‐Ouellet et al., 2011) and the pro‐inflammatory NF‐κB pathway (Shah et al., 2012), and immune system stimulation (Liu et al., 2014; Pacheco et al., 2004). Moreover, recent in vivo work from our group showed that constitutive genetic ablation of mGlu_5_ receptors in the SOD1^G93A^ mouse significantly increased survival probability and slowed the progression of the pathology (Bonifacino et al., 2017; Bonifacino, Provenzano, et al., 2019). Also there are several reports of mGlu5 receptors affecting a variety of cellular processes in ALS (Anneser et al., 1999; Brownell et al., 2015; Rossi et al., 2008; Vergouts et al., 2018; Vermeiren et al., 2006). Thus, considering their pleiotropic characteristics, mGlu_5_ receptors represent a promising and potential druggable target in CNS diseases (Ribeiro et al., 2017).

The simultaneous modulation of multiple mechanisms by mGlu_5_ receptors is relevant, considering that ALS is a multifactorial and multicellular disease and that each patient is likely to represent a unique clinical case that should be treated by targeting the appropriate etiological causes, even though that no relevant biomarkers are available at present for diagnosis. To overcome this impasse, several authors proposed the use of multimodal therapeutic approaches to interfere with multiple pathological mechanisms (Dorst et al., 2017). Apart from isolated cases that used a cocktail of drugs (Klivenyi et al., 2004; Kriz et al., 2003; Zhang et al., 2003) and a pioneering therapeutic attempt by combined gene silencing (Frakes et al., 2017), at present, there is no report of single drugs able to tackle different therapeutic targets. Therefore, the mGlu_5_ receptors represent an intriguing target to work on.

Several mGlu_5_ receptor antagonists or NAMs are available and have shown, in vitro, promising pharmacological effects, although they demonstrated unfavourable pharmacokinetic profiles, thus preventing a practical step towards in vivo treatments. In the last decade, there was a significant improvement, due to the synthesis of new compounds with favourable pharmacokinetics for in vivo application (Arsova et al., 2020). Based on our earlier data on the effects of the genetic down‐regulation of mGlu_5_ receptors and taking advantage of the availability of the new pharmacological tools, we designed a preclinical trial in SOD1^G93A^ mice using the oral bioavailable mGlu_5_ receptor NAM CTEP, a drug optimized for in vivo treatments in rodents (Lindemann et al., 2011), which has been already tested in mouse models of Huntington's, Parkinson's and Alzheimer's diseases (Abd‐Elrahman et al., 2017, 2018; Farmer et al., 2020).

Our experimental design included a first low‐dose trial with 2‐mg·kg^−1^ CTEP orally administered every 48 h, on the basis of the pharmacokinetic profile previously described by Lindemann et al. (2011). The therapeutic effects obtained by this first treatment did not match our expected results, increasing survival probability and a slowing down disease progression almost exclusively in the SOD1^G93A^ female group. Based on the limited, albeit significant, effects, we tested a higher dose of CTEP (4 mg·kg^−1^ every 24 h). The results obtained from the second trial turned out to be very encouraging because we observed a dose‐dependent amelioration of survival and clinical course in female mice and, most relevant, also in male mice.

Increasing the dose of CTEP differently affected the three aspects of motor ability we measured. In motor coordination, measured by rotarod and balance beam tests, no obvious CTEP dose dependency was observed in both males and females. On the contrary, a dose‐dependent amelioration of motor performance, measured by extension reflex and gait tests, and muscle force, measured by grip strength meter and hanging wire tests, was evident in SOD1^G93A^ males and females. The homogeneity of the response within the same test group and the different responses among the different test groups justify the need of a comprehensive battery of behavioural tests to systematically investigate the clinical aspects of the disease progression. CTEP showed a disease stage‐specific effect selectively in hanging wire and grip strength meter tests, but this effect was observed only during the first phase of treatment. Interestingly, supporting this latter aspect, Abd‐Elrahman et al. reported that CTEP effectively reverses cognitive deficits in a mouse model of Alzheimer's disease after 24‐week treatment. When treatment was extended to 36 weeks, the drug no longer mitigated the disease symptoms (Abd‐Elrahman et al., 2020).

One main finding of the present investigation is the sex‐related actions of CTEP, which primarily affects disease progression in females. To shed light on the possible causes of this difference, we performed a comprehensive series of experiments to measure MN number, astrogliosis and microgliosis in the ventral horns of lumbar spinal cord and the tissue distribution of CTEP, examining vehicle‐ and CTEP‐treated WT and SOD1^G93A^ male and female mice. However, we found no obvious differences in the recovery from MN loss, astrogliosis and microgliosis after CTEP treatment comparing the two sexes. Also, the pharmacokinetic studies indicated that CTEP accesses the brain equally well in male and female WT and SOD1^G93A^ mice. How comparable effects on cellular disease markers and similar CTEP distribution in SOD1^G93A^ mice could reflect different impacts of CTEP on the disease progression in males and females is difficult to explain. This issue becomes even more complex because we have already seen sex‐selective responses after in vivo genetic ablation of mGlu_5_ receptors in SOD1^G93A^ mice, with the effects more pronounced in male mice (Bonifacino et al., 2017; Bonifacino, Provenzano, et al., 2019). Of note, sex‐specific male‐oriented effects have been described also in a CTEP‐treated mouse model of Alzheimer's disease (Abd‐Elrahman et al., 2019). To conclude, other mechanisms should be investigated to shed light on the differences here observed, also focussing on the pharmacodynamic aspects linked to mGlu_5_ receptor expression, distribution and function at different disease stages.

In any case, MN death was considerably decreased after CTEP at both the low and high doses. This outcome is particularly remarkable, considering that the treatment started at Day 90, whereas MN damage and death is an early phenomenon that takes place well in advance of the beginning of the treatment (Dukkipati et al., 2018). Damage of MNs in ALS is a non‐cell autonomous event, in fact astrocytes and microglia play a pivotal role in MN vulnerability (Filipi et al., 2020). We observed here a reduction of astrogliosis and microgliosis after CTEP treatment, which conceivably ameliorates the noxious milieu and protects MNs, reducing the disease severity.

Ex vivo functional experiments showed that, in vivo CTEP chronic treatment reduced the excessive glutamate release evoked by high KCl or mGlu_1/5_ receptor activation, supporting the main role played by excessive glutamate neurotransmission in ALS and the involvement of mGlu_5_ receptors in this phenomenon. If this was the case, one may wonder why riluzole, whose proposed chief mechanism is to reduce glutamate release‐blocking voltage‐gated Na^+^ channels, presents only limited therapeutic efficacy. However, lessening Na^+^ channel permeability, riluzole non‐specifically can inhibit the release of both excitatory and inhibitory neurotransmitters. A more direct modulation of excitatory transmission could increase the efficiency of the therapies.

The results here obtained support the view that the selective modulation of mGlu_5_ receptors represents an effective therapeutic approach, with clinical potential, to ALS. Of note, other mGlu_5_ receptor NAMs have been tested in human clinical studies for the treatment of fragile X syndrome, depression, levodopa‐induced dyskinesia and Huntington's disease (Reilmann et al., 2015; Trenkwalder et al., 2016). In particular, basimglurant, an imidazole derivative analogue of CTEP, has been optimized for human studies with very favourable pharmacokinetic and toxicological facets (Jaeschke et al., 2015; Lindemann et al., 2015). Basimglurant has been successfully tested in clinical trials for the cure of depression and fragile X syndrome (Quiroz et al., 2016; Youssef et al., 2018). At present, there have been no clinical attempts to test mGlu_5_ receptor NAMs in ALS and our data represents the first preclinical evidence supporting the utility of these drugs as new tools for the treatment of ALS.

## AUTHOR CONTRIBUTIONS

B.G., M.M. and B.T. contributed to the conception and design of the project; T.C., K.M. and N.T.P.N. performed the chronic treatments with CTEP; P.F., Z.A.R., F.G. and B.M. performed the behavioural experiments; T.C., R.S., B.N. and F.S. performed the histological and immunohistogical experiments; V.M. and P.A. measured the CTEP concentration in the brain, liver and plasma; B.G., M.M., T.B., R.S. and V.M. contributed to the analysis and interpretation of the data; B.G., M.M. and T.B. contributed to writing and revising the manuscript; and all authors read and approved the final manuscript.

## CONFLICT OF INTEREST

The authors declare no conflicts of interest.

## DECLARATION OF TRANSPARENCY AND SCIENTIFIC RIGOUR

This Declaration acknowledges that this paper adheres to the principles for transparent reporting and scientific rigour of preclinical research as stated in the *BJP* guidelines for Design & Analysis, Immunoblotting and Immunochemistry, and Animal Experimentation and as recommended by funding agencies, publishers and other organizations engaged with supporting research.

## Supporting information

**Figure S1.** Cytoplasmic vacuoles in spinal cord motor neurons of WT, vehicle and CTEP‐treated SOD1^G93A^ mice.
The presence of cytoplasmic vacuoles has been assessed in ventrolateral horn of L4‐L5 spinal cord sections from WT, vehicle‐treated and CTEP‐treated (4 mg/kg/24 h) mixed sex mice. Representative 2x magnification light microscopy images (scale bar 100 μm) spinal cord slices after haematoxylin & eosin staining, from WT, vehicle‐treated SOD1^G93A^ and CTEP treated SOD1^G93A^ mice are reported. Red arrows indicate cytoplasmic vacuoles.

**Table S1.** Score scale for the evaluation of the hind limb extension reflex and gait impairment in SOD1^G93A^ mice.

**Table S2.** Median survival age values (days) for vehicle‐treated and CTEP‐treated SOD1^G93A^ mice.

**Data S1.** Supporting Information

## Data Availability

Data are available on request from the authors.
