# Supplementary figures and images for: Blocking glutamate mGlu5 receptors with the negative allosteric modulator CTEP improves disease course in SOD1G93A mouse model of amyotrophic lateral sclerosis
Source: Br J Pharmacol. 2021 Jun 29;178(18):3747–64. doi: 10.1111/bph.15515 (PMC8457068; doi:10.1111/bph.15515)

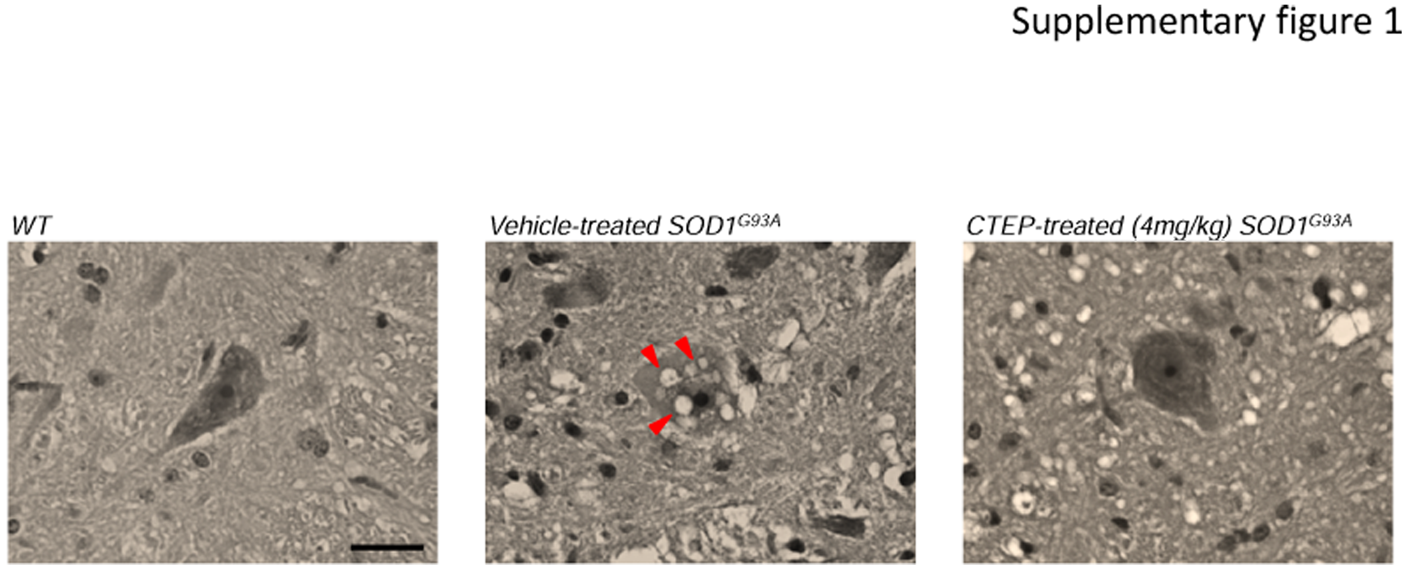

Supplement: Supplementary file 1 — Figure S1. Cytoplasmic vacuoles in spinal cord motor neurons of WT, vehicle and CTEP‐treated SOD1G93A mice. The presence of cytoplasmic vacuoles has been assessed in ventrolateral horn of L4‐L5 spinal cord sections from WT, vehicle‐treated and CTEP‐treated (4 mg/kg/24 h) mixed sex mice. Representative 2x magnification light microscopy images (scale bar 100 μm) spinal cord slices after haematoxylin & eosin staining, from WT, vehicle‐treated SOD1G93A and CTEP treated SOD1G93A mice are reported. Red arrows indicate cytoplasmic vacuoles. [file BPH-178-3747-s002.tif]

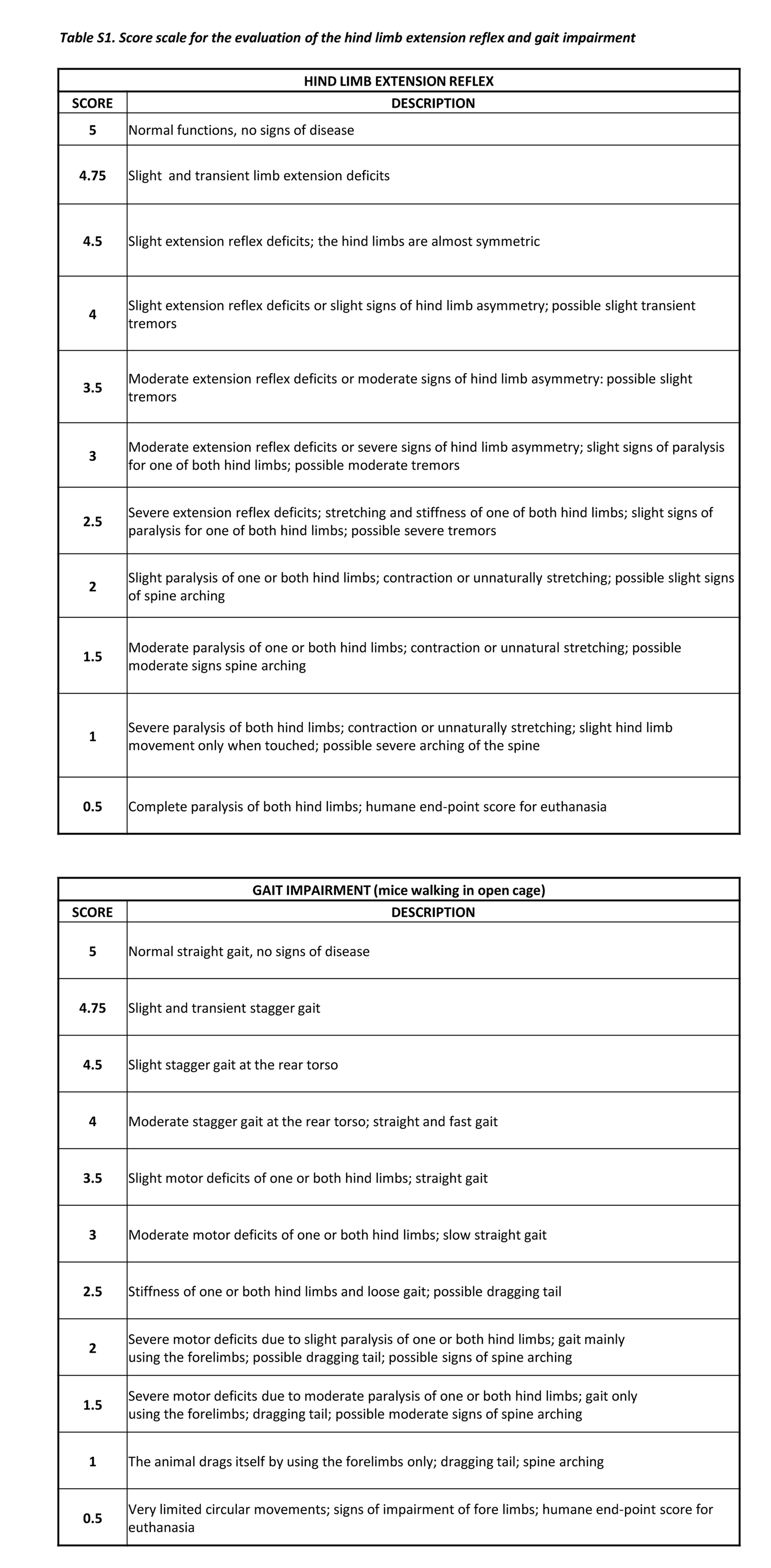

Supplement: Supplementary file 2 — Table S1. Score scale for the evaluation of the hind limb extension reflex and gait impairment in SOD1G93A mice. [file BPH-178-3747-s004.tif]

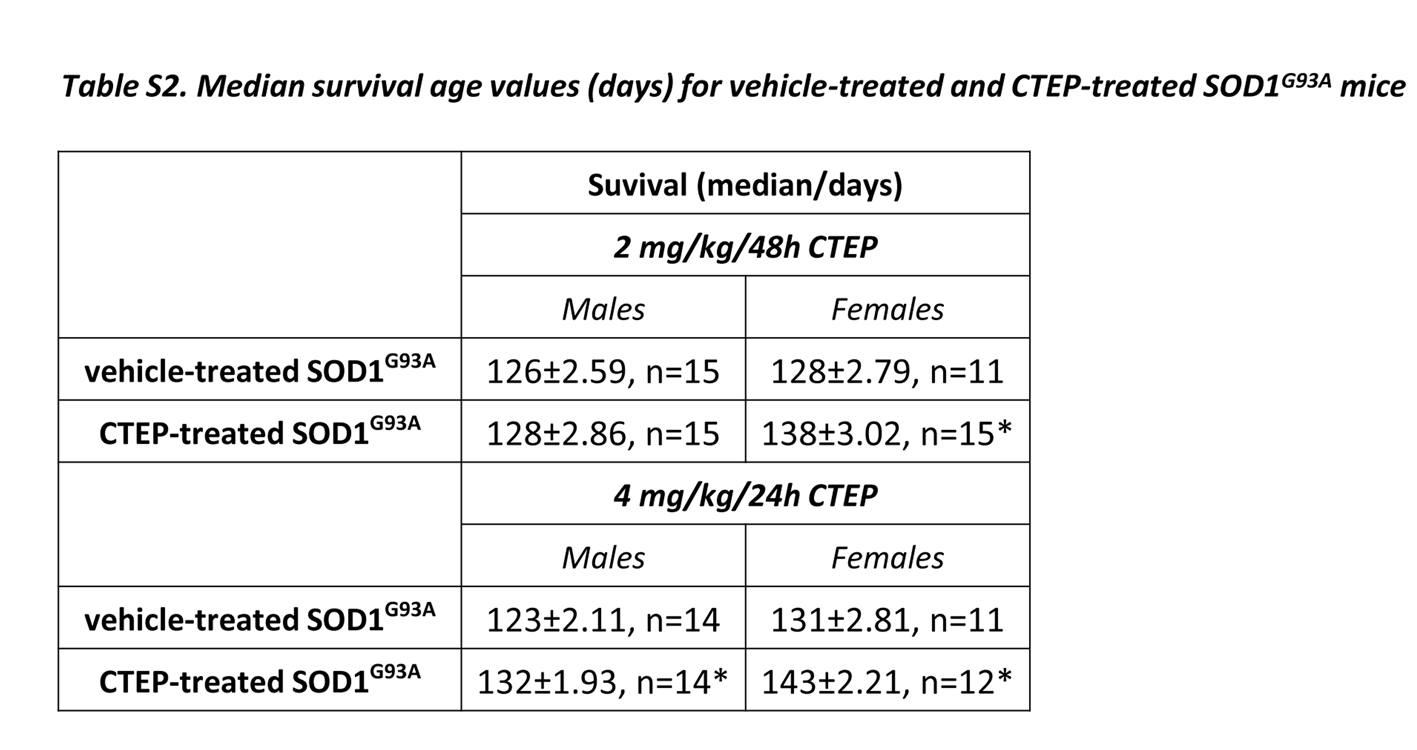

Supplement: Supplementary file 3 — Table S2. Median survival age values (days) for vehicle‐treated and CTEP‐treated SOD1G93A mice. [file BPH-178-3747-s001.tif]
