# Supplementary material for: Blocking glutamate mGlu5 receptors with the negative allosteric modulator CTEP improves disease course in SOD1G93A mouse model of amyotrophic lateral sclerosis
Source: Br J Pharmacol. 2021 Jun 29;178(18):3747–64. doi: 10.1111/bph.15515 (PMC8457068; doi:10.1111/bph.15515)

Supporting material for Reviewers only

Supporting material for Reviewers only
(biological replicates used for histological MN counts, in male treated mice at the low dose of CTEP 2mg/kg/48h)

Supporting material at the figure 6

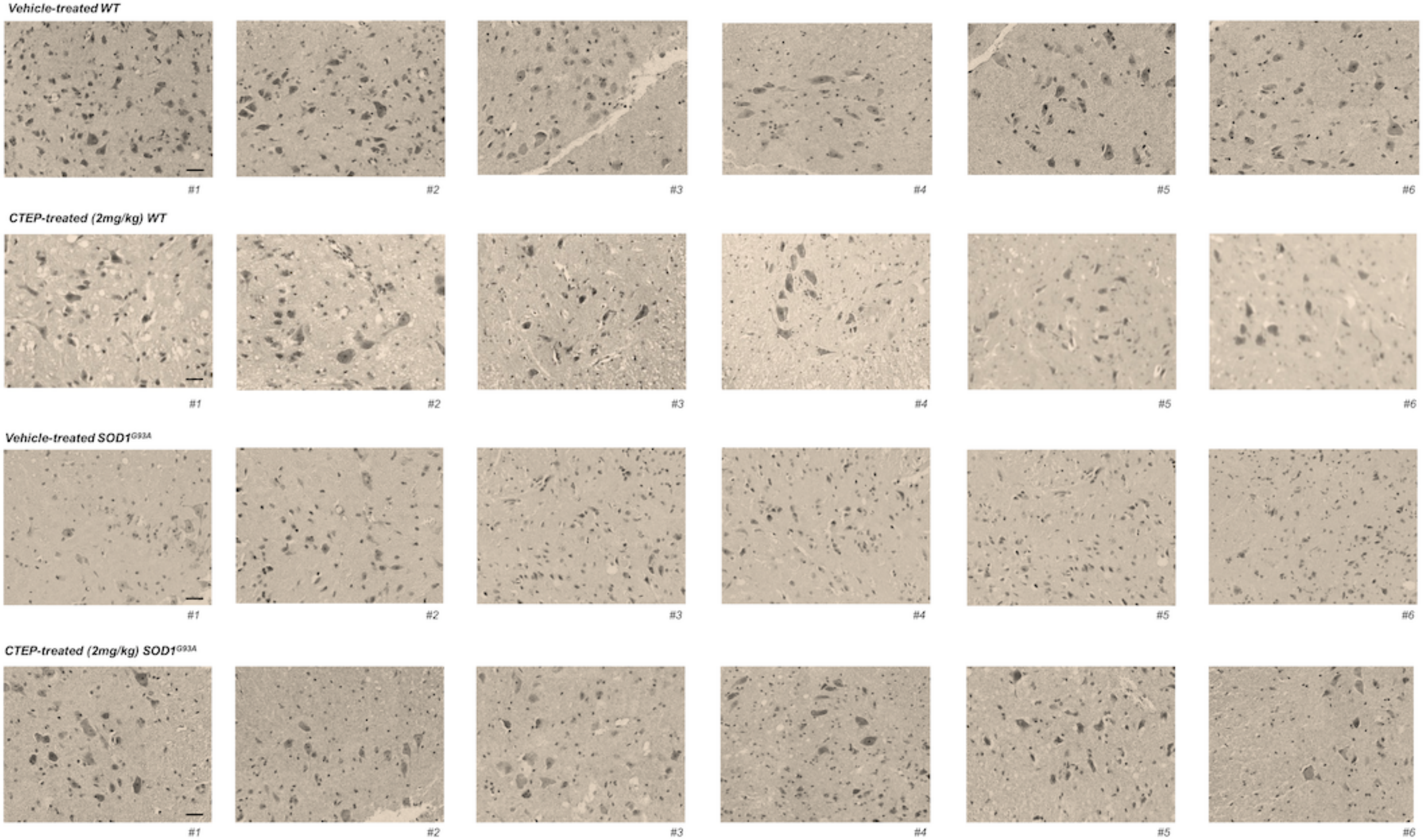

Supporting material at the figure 6

Supporting material for Reviewers only
(biological replicates used for histological MN counts, in female treated mice at the high dose of CTEP 2mg/kg/48h)

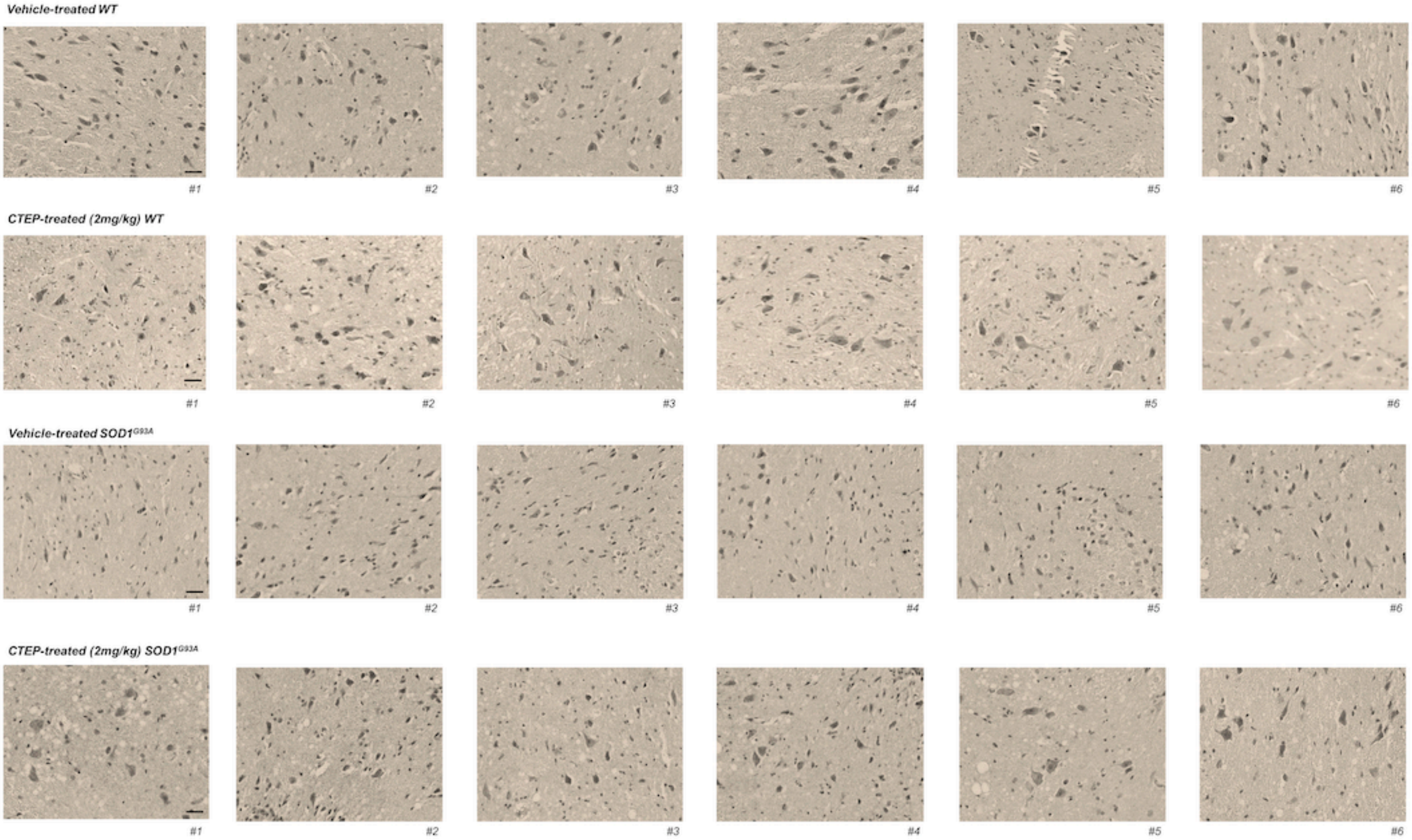

Supporting material at the figure 6

Supporting material for Reviewers only
(biological replicates used for histological MN counts, in male treated mice at the high dose of CTEP 4mg/kg/24h)

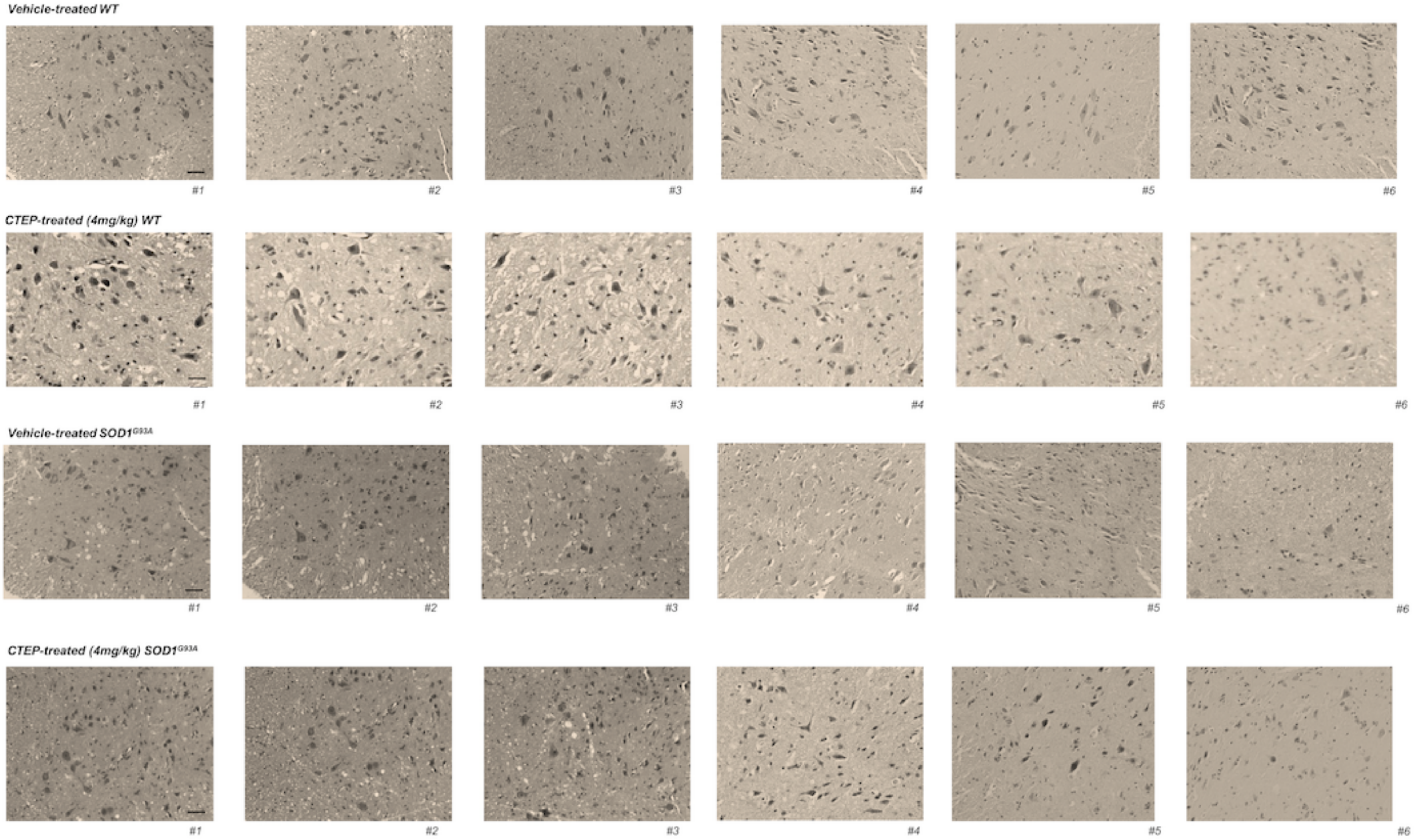

Supporting material at the figure 6

Supporting material for Reviewers only
(biological replicates used for histological MN counts, in female treated mice at the high dose of CTEP 4mg/kg/24h)

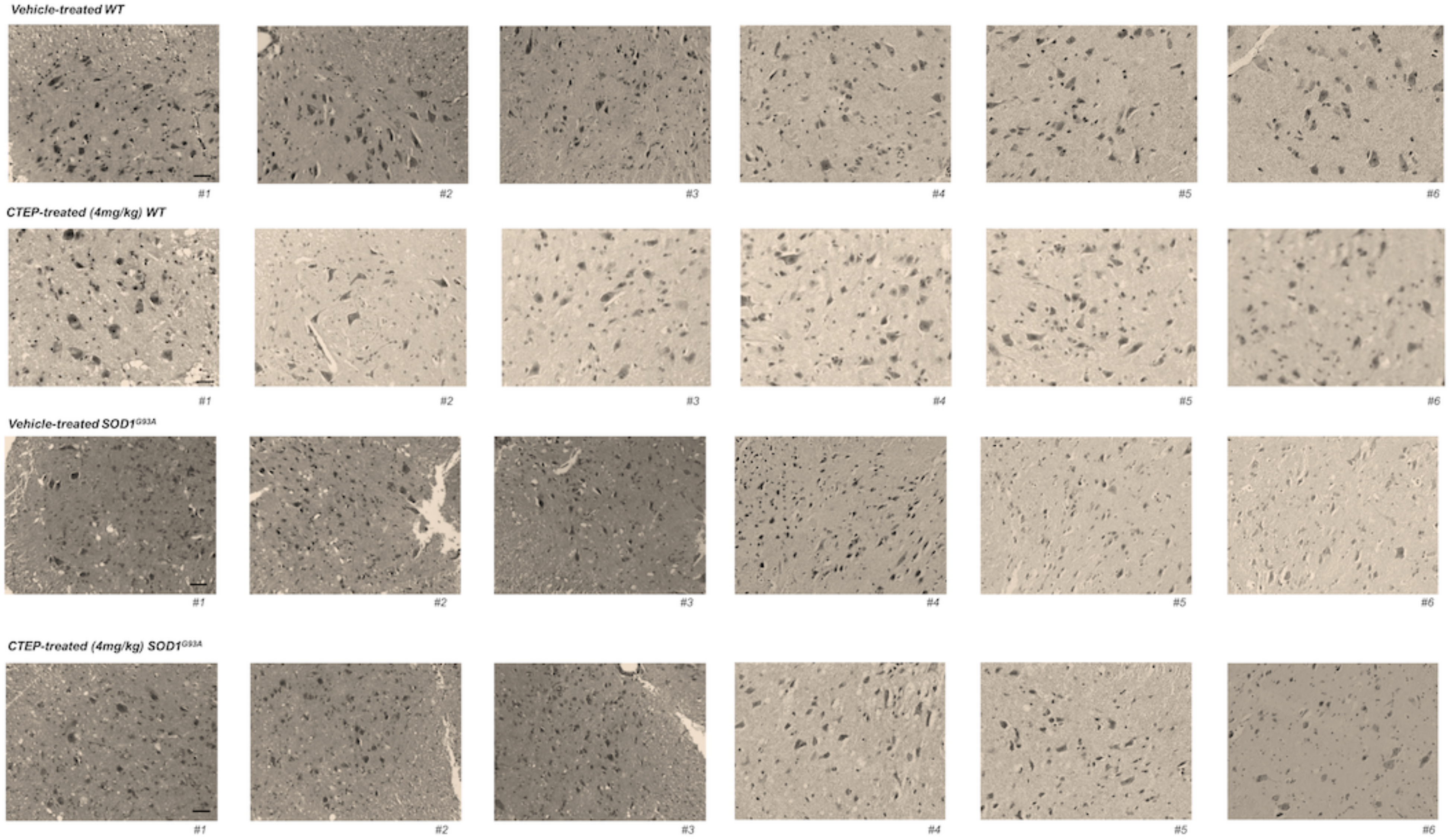

Supporting material for Reviewers only

Supporting material at the figure 6

(biological replicates used for quantification analyses of GFAP immunostaining, in male treated mice at the low dose of CTEP 2mg/kg/48h)

Vehicle-treated WT

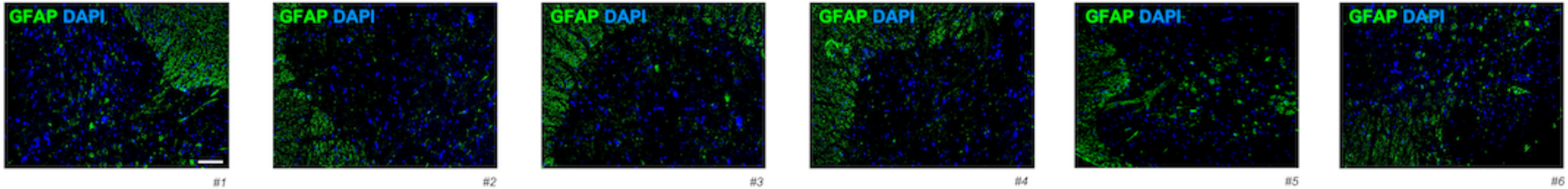

CTEP-treated (2mg/kg) WT

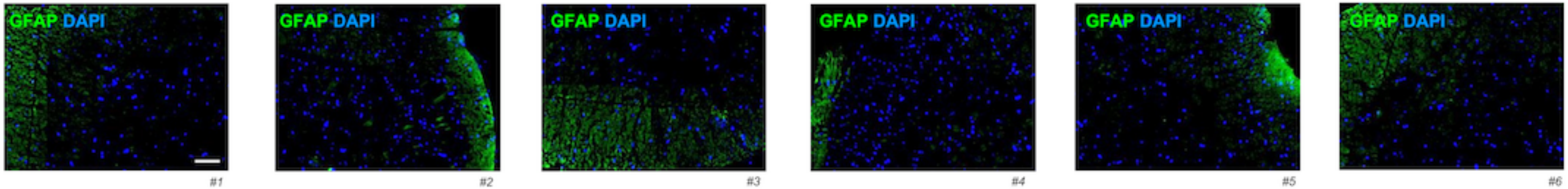

Vehicle-treated SOD1^{G93A}

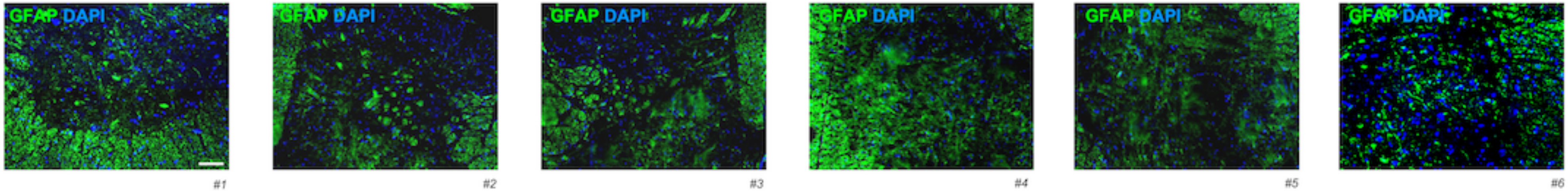

CTEP-treated (2mg/kg) SOD1^{G93A}

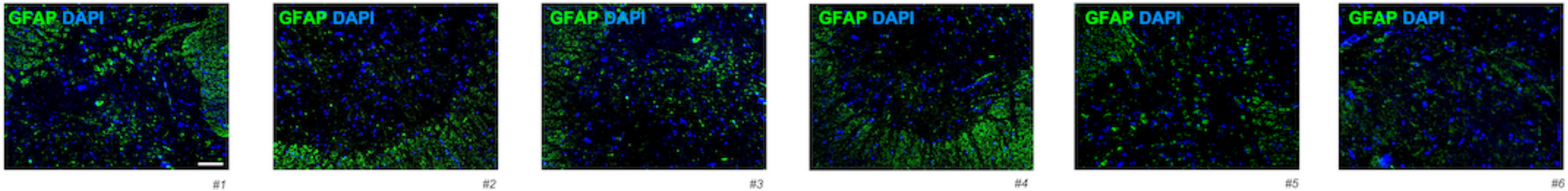

Supporting material at the figure 6

Supporting material for Reviewers only
(biological replicates used for quantification analyses of IBA1 immunostaining, in female treated mice at the low dose of CTEP 2mg/kg/48h)

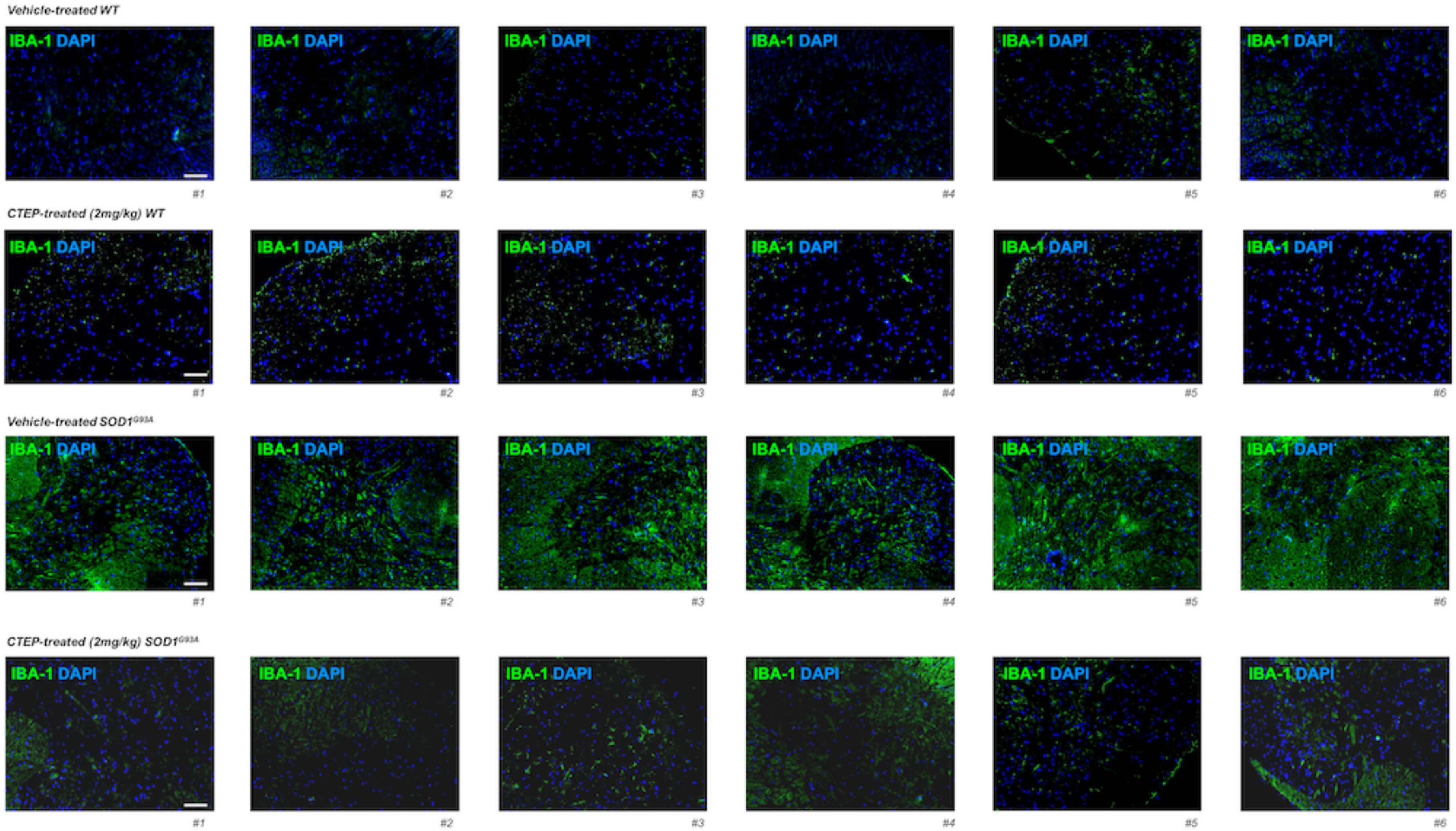

Supporting material at the figure 6

Supporting material for Reviewers only
(biological replicates used for quantification analyses of GFAP immunostaining, in male treated mice at the high dose of CTEP 4mg/kg/24h)

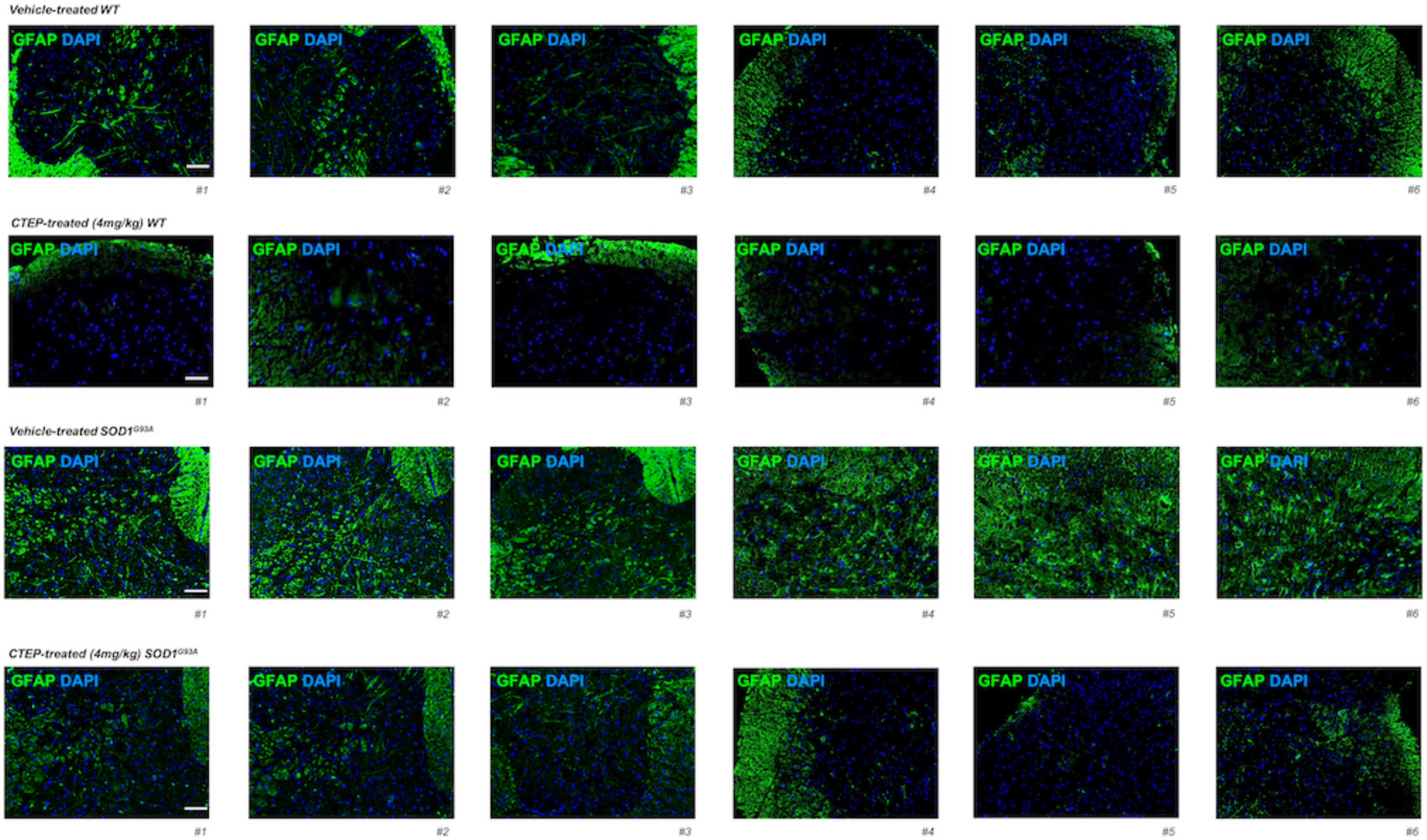

Supporting material at the figure 6

Supporting material for Reviewers only
(biological replicates used for quantification analyses of GFAP immunostaining, in female treated mice at the high dose of CTEP 4mg/kg/24h)

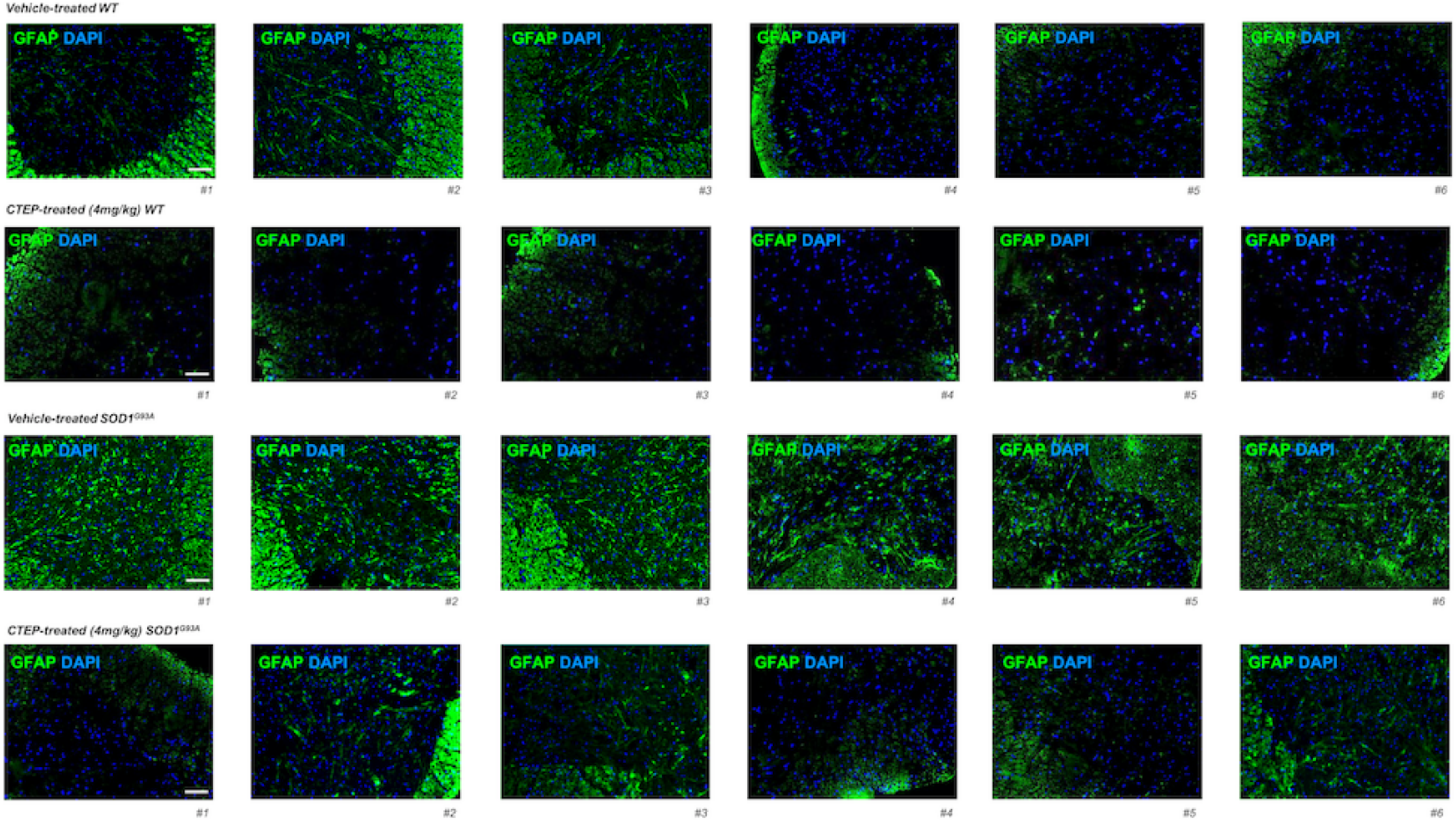

Supporting material at the figure 6

Supporting material for Reviewers only
(biological replicates used for quantification analyses of IBA1 immunostaining, in male treated mice at the low dose of CTEP 2mg/kg/48h)

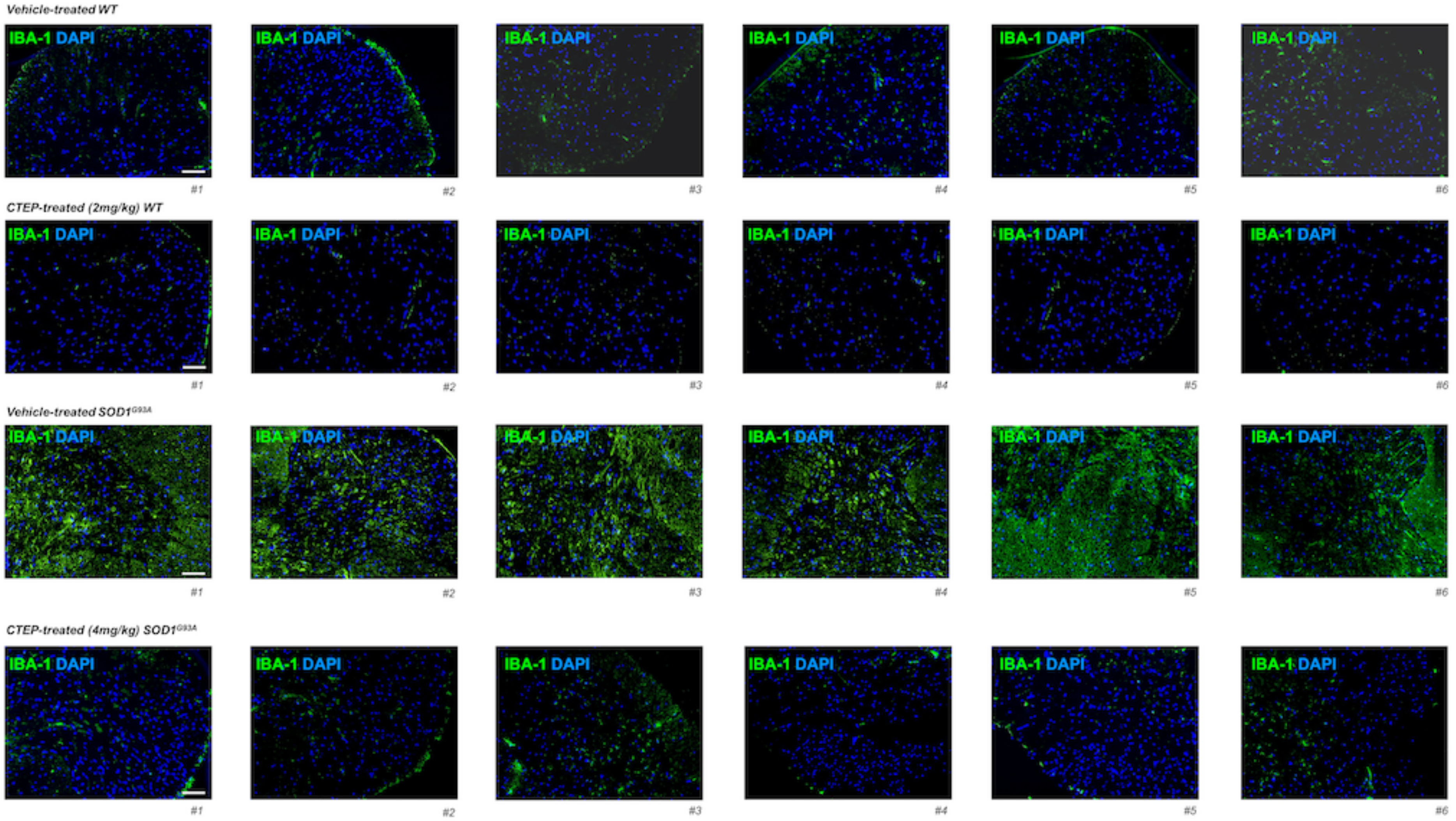

Supporting material at the figure 6

Supporting material for Reviewers only
(biological replicates used for quantification analyses of IBA1 immunostaining, in female treated mice at the low dose of CTEP 2mg/kg/48h)

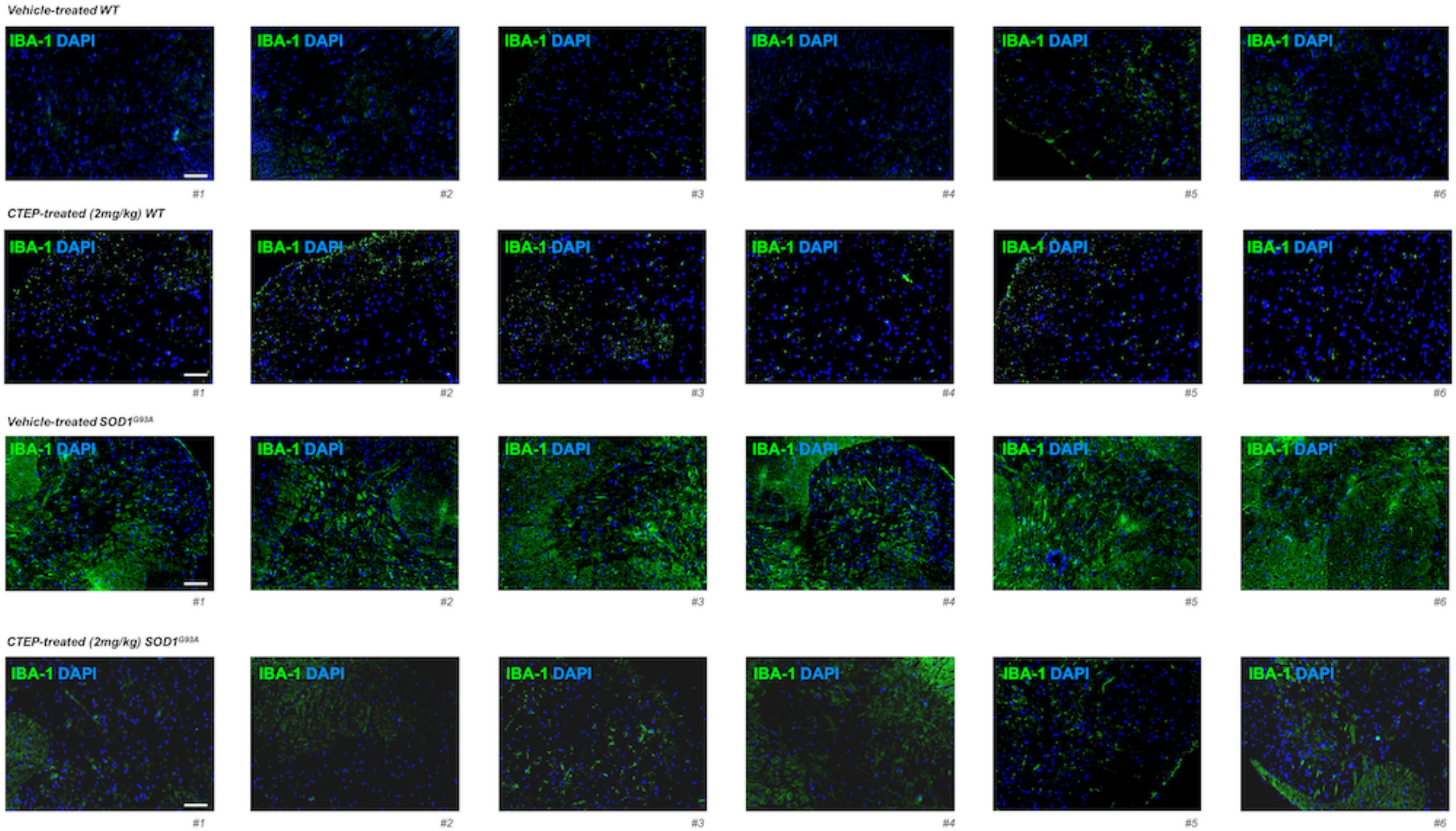

Supporting material at the figure 6

Supporting material for Reviewers only
(biological replicates used for quantification analyses of IBA1 immunostaining, in male treated mice at the high dose of CTEP 4mg/kg/24h)

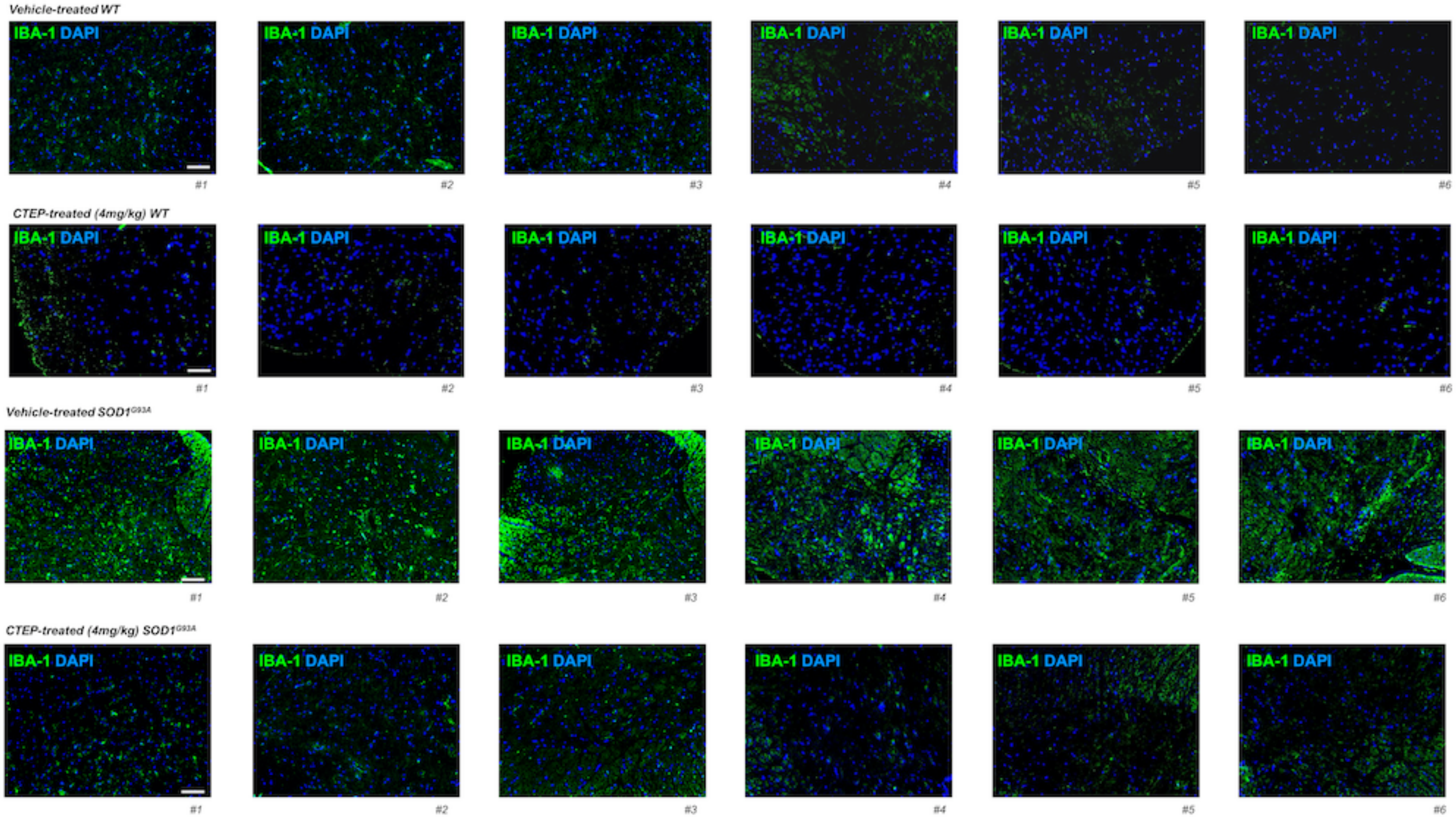

Supporting material at the figure 6

Supporting material for Reviewers only
(biological replicates used for quantification analyses of IBA1 immunostaining, in female treated mice at the high dose of CTEP 4mg/kg/24h)

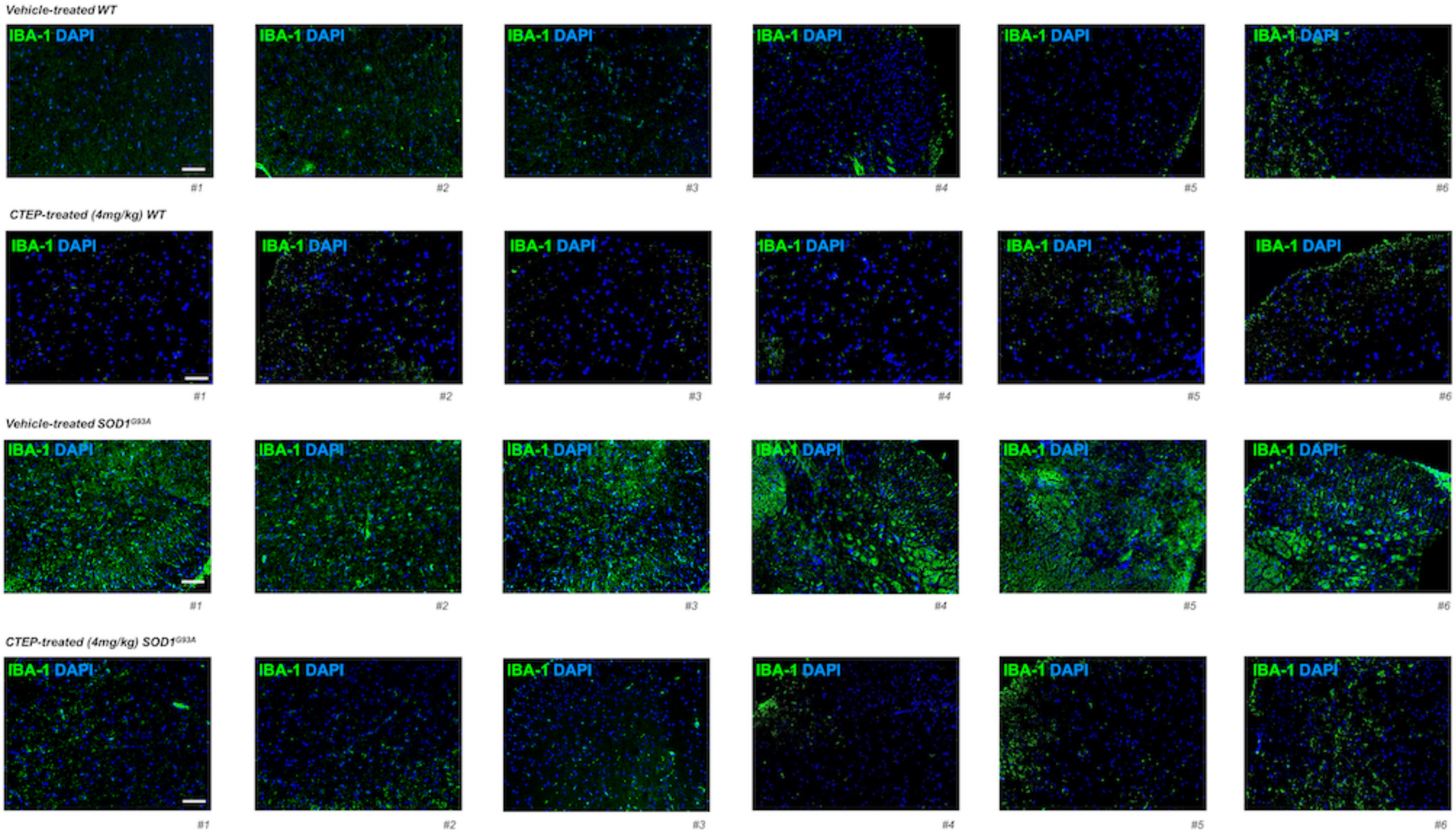

Supplement: Supplementary file 4 — Data S1. Supporting Information [file BPH-178-3747-s003.pdf]
